# KLF5 Downregulation Links Impaired BNIP3‐Mediated Mitophagy to Inflammatory Valve Remodeling in Calcific Aortic Valve Disease

**DOI:** 10.1002/advs.77368

**Published:** 2026-08-27

**Authors:** Jin‐Hui Bian, Jia‐Xi Gu, Zhang Yue, Chun‐Ze Yuan, Hai‐Bin Ji, Ao Li, Yu Zhong, Xiu‐Fan Xu, Yu‐Qiu He, Ye‐Fan Jiang, Hong‐Shan Chen, Xiang Chen, Meng‐Lin Chao, Yong‐Feng Shao

**Affiliations:** ^1^ Department of Cardiovascular Surgery The First Affiliated Hospital of Nanjing Medical University Nanjing China; ^2^ Key Laboratory of Drug Targets and Translational Medicine For Cardio‐cerebrovascular Diseases School of Pharmacy Nanjing Medical University Nanjing China; ^3^ Department of Laboratory Medicine Yancheng No. 1 People's Hospital Affiliated Hospital of Medical School Nanjing University Yancheng China

**Keywords:** BNIP3, calcific aortic valve disease, Krüppel‐like factor 5, mitochondrial DNA

## Abstract

Calcific aortic valve disease (CAVD) lacks effective drug therapy. This study integrated human valve transcriptomic datasets and tissue specimens, public single‐cell and spatial transcriptomics, primary human valvular interstitial cell (VIC) models, and ApoE^−/−^ mice fed a high‐fat diet to define the role of KLF5 in VIC remodeling. KLF5 expression was reduced in calcified human valves and associated with hemodynamic severity; tissue and single‐cell analyses localized this reduction to VIC‐rich lesions and disease‐associated VIC states. In primary VICs, KLF5 overexpression suppressed, whereas KLF5 knockdown enhanced, osteogenic medium‐induced inflammatory signaling and mineralization. Systemic AAV‐mediated KLF5 overexpression attenuated valve dysfunction, leaflet thickening, mineral deposition, and BMP2 expression in ApoE^−/−^ mice. Mechanistically, KLF5 loss increased cytosolic mtDNA accumulation and activated cGAS–STING and NLRP3‐associated signaling, whereas cGAS knockdown reduced downstream signaling and interferon‐stimulated gene induction. KLF5 directly activated the BNIP3 promoter and supported BNIP3‐mediated mitophagy. BNIP3 knockdown markedly reduced the ability of KLF5 overexpression to preserve mitophagic flux and restrain inflammatory signaling and mineralization, whereas BNIP3 re‐expression mitigated the corresponding phenotypes in KLF5‐deficient VICs. These findings identify a VIC‐centered KLF5–BNIP3 regulatory pathway that supports mitochondrial quality control and limits mtDNA‐sensitive sterile inflammation, providing a mechanistic rationale for future therapeutic investigation in CAVD.

## Graphical abstract

In human CAVD, KLF5 is reduced in VIC‐rich regions and remodeling/stress‐associated VIC states. In VICs, KLF5 sustains BNIP3 promoter activity and BNIP3‐mediated mitophagy, thereby limiting cytosolic mtDNA accumulation. KLF5 loss weakens mitochondrial quality control and enhances mtDNA‐sensitive STING/NF‐κB/NLRP3 inflammatory signaling under osteogenic stress, amplifying inflammatory signaling and mineralization. Spatial transcriptomics localizes this KLF5‐low DAVIC program to inflammatory–osteogenic lesions in calcified valves.

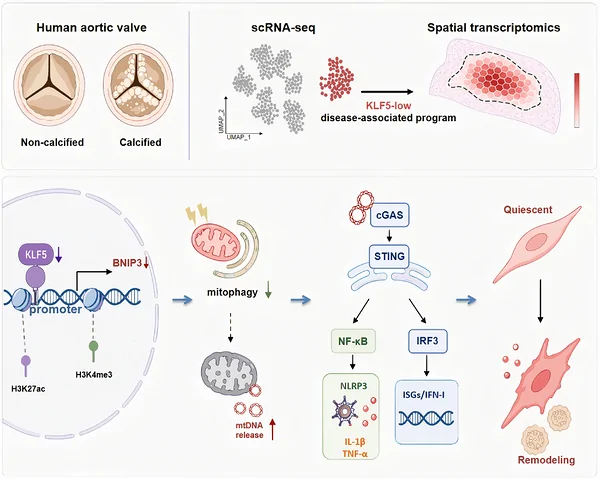

**Abbreviations**
3C‐qPCR: Chromosome conformation capture‐qPCR
AAV2: Adeno‐associated virus subtype 2
AAV‐KLF5: adeno‐associated virus carrying KLF5 overexpression vector
AAV‐scr: adeno‐associated virus carrying scramble control vector
BNIP3: BCL2 and adenovirus E1B 19‐kDa‐interacting protein 3
ChIP: chromatin immunoprecipitation
CAVD: Calcific aortic valve disease
cGAS–STING: cyclic GMP–AMP synthase–stimulator of interferon genes
DHE: Dihydroethidium
DAVIC: disease‐associated valvular interstitial cell
EtBr: ethidium bromide
HFD: high‐fat diet
ISGs: interferon‐stimulated genes
KLF5: Krüppel‐like factor 5
mtDNA: mitochondrial DNA
NF‐κB: nuclear factor‐κB
OM: osteogenic medium
qRT‐PCR: Quantitative real‐time polymerase chain reaction
ROS: Reactive oxygen species
siRNA: small interfering RNA
TEM: Transmission electron microscopy
TSS: transcription start site
VSMCs: vascular smooth muscle cells
VIC: valvular interstitial cell

## 1 Introduction

Calcific aortic valve disease (CAVD) is the most common valvular heart disease in older adults, and its prevalence increases markedly with age [1]. Progressive leaflet thickening, extracellular matrix remodeling, and calcification ultimately lead to aortic stenosis, adverse ventricular remodeling, and heart failure [2]. Despite its clinical burden, no pharmacological therapy has been shown to prevent or reverse calcific valve remodeling [3]. Surgical or transcatheter valve replacement remains the only definitive treatment for advanced disease, but these interventions address end‐stage valve obstruction rather than the cellular and molecular mechanisms that initiate and propagate valve pathology [4]. This therapeutic gap highlights the need to define the cellular and molecular mechanisms that drive disease progression.

CAVD is an active fibro‐inflammatory and osteogenic disease involving endothelial dysfunction, lipid deposition, extracellular matrix remodeling, immune activation, and phenotypic changes in resident valve cells [5, 6]. Valvular interstitial cells (VICs), the predominant stromal population of the valve leaflet, maintain extracellular matrix homeostasis under physiological conditions but acquire activated, inflammatory, and osteogenic states under pathological stress. Single‐cell and multi‐omic studies have further resolved disease‐associated VIC states and cell‐type‐specific inflammatory, metabolic, and matrix‐remodeling programs in calcified valves [7, 8]. However, the transcriptional mechanisms that maintain VIC homeostasis and limit maladaptive inflammatory–osteogenic remodeling remain incompletely defined.

Inflammation has emerged as a key driver of cardiovascular diseases, including atherosclerosis, heart failure, and vascular remodeling, and is increasingly recognized in CAVD pathophysiology [9, 10]. Prolonged inflammatory activation can promote osteogenic remodeling of VICs, contributing to calcific valve pathology [5]. Prior studies have mainly focused on canonical inflammatory pathways such as nuclear factor‐κB (NF‐κB) and the NLRP3 inflammasome [11, 12], but the mechanisms linking disease‐associated VIC remodeling to sterile inflammatory signaling and osteogenic activation have not been established.

The cyclic GMP–AMP synthase–stimulator of interferon genes (cGAS–STING) pathway has emerged as a cytosolic double‐stranded DNA‐sensing axis that activates type I interferon and downstream inflammatory cascades [13, 14]. In heart failure, cardiac hypertrophy, and other cardiovascular conditions, impaired mitochondrial quality control results in the cytosolic release of mitochondrial DNA (mtDNA), which subsequently activates cGAS–STING signaling [15, 16, 17]. Mitochondrial damage and mtDNA release have also been linked to cGAS–STING activation and valve calcification in CAVD [18]. These findings implicate cGAS–STING signaling in calcific valve remodeling. Nevertheless, the upstream transcriptional control of mitochondrial quality control and mtDNA‐dependent inflammatory activation in disease‐associated VIC states is poorly characterized. Early‐onset cardiovascular calcification in Singleton–Merten syndrome further supports a broader association between sustained type I interferon activation and calcific cardiovascular disease [19].

Mitochondrial homeostasis is central to cellular health, and mitophagy, the selective clearance of damaged mitochondria, plays a crucial role in maintaining this balance [20]. Defective mitophagy leads to mitochondrial dysfunction, mtDNA leakage, and activation of inflammatory pathways, including cGAS–STING [21, 22]. In hypertrophic cardiomyopathy, loss of PINK1‐mediated mitophagy promotes mtDNA release and cGAS–STING–dependent inflammation [15]. BCL2 and adenovirus E1B 19‐kDa‐interacting protein 3 (BNIP3), a receptor‐mediated mitophagy regulator, has been implicated in cardiovascular stress adaptation, including ischemic heart disease and dilated cardiomyopathy [23, 24]. Impaired mitophagy has also been observed in CAVD [25], suggesting that defective mitochondrial clearance may contribute to inflammatory signaling and mineralization in VICs. However, the transcriptional control of BNIP3‐mediated mitophagy in VICs has not been resolved.

Krüppel‐like factors (KLFs) are zinc‐finger transcription factors that regulate vascular homeostasis, inflammation, metabolism, and cellular stress responses [26, 27, 28]. KLF5 has been implicated in cardiovascular inflammation, mitochondrial function, vascular remodeling, and calcification‐related transcriptional programs [29, 30]. In vascular smooth muscle cells (VSMCs), KLF5 promotes phosphate‐induced calcification through transcriptional activation of RUNX2 [31]. Whether KLF5 regulates mitochondrial quality control and mtDNA‐sensitive inflammatory signaling in VICs has not been established.

The present study combined human valve transcriptomic datasets and tissue specimens, public single‐cell and spatial transcriptomic analyses, primary human VIC experiments, and a hyperlipidemic mouse model to examine the role of KLF5 in VIC remodeling and mitochondrial inflammatory signaling in CAVD.

## 2 Materials and Methods

### 2.1 Human Aortic Valve Collection

Human aortic valve tissues were obtained from patients undergoing aortic valve replacement for CAVD or patients undergoing Bentall procedures or heart transplantation, with valves from the latter procedures serving as non‐calcified references. Non‐calcified reference valves were tricuspid aortic valves without macroscopic calcific nodules; pre‐operative echocardiography showed no significant stenosis, and intra‐operative inspection confirmed three distinct cusps/commissures without raphe or commissural fusion. One leaflet per case was processed for histology; Alizarin Red staining was used to verify the absence of calcium deposits in reference valves and presence in CAVD specimens. CAVD samples were obtained from patients with clinical aortic stenosis, macroscopic calcific nodules, and positive Alizarin Red staining on the resected leaflet. Exclusion criteria included congenital bicuspid aortic valve and functional bicuspidization (acquired bicuspid–like anatomy due to commissural fusion), rheumatic valve disease, infective endocarditis, and systemic autoimmune disorders. Case adjudication followed a two‐step workflow: (i) pre‐operative echocardiographic assessment confirming a tricuspid aortic valve with three cusps/commissures and no raphe or commissural fusion; (ii) intra‐operative inspection by the operating surgeon confirming three distinct cusps and commissures. Any specimen with a raphe, commissural fusion, or indeterminate morphology was excluded. Only leaflet tissue was collected; no aortic root tissue was collected. The clinical characteristics of patients providing non‐calcified reference valves and patients with CAVD are shown in Table S1. All patients provided informed consent prior to inclusion, and the study was conducted in accordance with the Declaration of Helsinki and approved by the Ethics Committee of the First Affiliated Hospital of Nanjing Medical University (ethical approval number: 2019‐SRFA‐280).

### 2.2 Cell Culture and Treatment

Primary human VICs were isolated from non‐calcified aortic valve leaflets obtained during Bentall procedures or heart transplantation using a previously described collagenase method [32]. Valve leaflets were first washed in phosphate‐buffered saline (PBS; Gibco, C10010500BT), then digested in a collagenase I solution (2.5 mg/mL; ThermoFisher Scientific, 17100017) at 37°C for 40 min to remove surface endothelial cells. Following this, fresh collagenase solution (2 mg/mL) was used for further digestion at 37°C for 3 to 8 h. Following centrifugation at 100×g for 10 min, cells were resuspended and cultured in Dulbecco's Modified Eagle's Medium (DMEM; Gibco, 11995065) supplemented with 10% fetal bovine serum at 37°C in a 5% CO_2_ atmosphere. VICs from passages 3 to 6 were used in subsequent experiments. VICs were treated with osteogenic medium (OM) to induce osteogenic differentiation, prepared as previously described [33]. To investigate the role of mtDNA, primary human VICs were treated with 50 ng/mL ethidium bromide (EtBr) to induce mtDNA depletion, with medium changes every 48 h. The efficiency of depletion was verified by qPCR analysis of mitochondrial‐encoded genes (MT‐ND1 and MT‐ND2).

### 2.3 Animal Models and Treatment

All animal experiments were conducted in accordance with the guidelines approved by the Institutional Animal Care and Use Committee of Nanjing Medical University (ethical approval number: IACUC‐2210022). Six‐week‐old male ApoE^−/−^ mice were purchased from Changzhou Kavins Experimental Animal Co., Ltd. and housed under specific pathogen‐free conditions. Mice were randomly assigned to the high‐fat diet (HFD) or normal diet (ND; standard chow) groups. To induce aortic valve calcification, mice in the HFD group were fed a diet containing 40% kcal from fat for 20 weeks, while control mice received a normal diet. To investigate KLF5 function, ND‐ and HFD‐fed mice were injected via tail vein with Adeno‐associated virus subtype 2 (AAV2) carrying either the KLF5 overexpression vector (AAV‐KLF5) or a negative control vector, AAV‐scramble (AAV‐scr). Subsequently, mice were lightly anesthetized with 1.5% isoflurane, and echocardiographic parameters were assessed using the Vevo 2100 imaging system (VisualSonics, Toronto, Canada). Mice were then euthanized by intraperitoneal injection of sodium pentobarbital (200 mg/kg), and cardiac tissues were collected for subsequent histological analysis, including H&E staining, Von Kossa staining, and immunofluorescence staining.

### 2.4 Alizarin Red Staining

Alizarin Red staining was performed as described previously [34]. When cells reached approximately 80% confluence, VICs were cultured in osteogenic medium supplemented with 10 mM β‐glycerophosphate (Sigma–Aldrich, G9422), 100 nM dexamethasone (Sigma–Aldrich, D4902), and 50 µg/mL L‐ascorbic acid (Sigma–Aldrich, A4544) for 21 days, with medium changes every 3 days. To assess calcium deposition, cells were stained with Alizarin Red solution using a commercial staining kit according to the manufacturer's instructions. Briefly, cells were washed, fixed with 4% paraformaldehyde for 15 min, and then incubated with 0.2% Alizarin Red solution (Beyotime Biotechnology, C0138) for 30 min. Excess dye was removed, and the stained samples were documented using a Nikon ST2 microscope (Japan). Alizarin Red–positive area was quantified using ImageJ (NIH, Bethesda, MD, USA) using identical settings/thresholds across groups within each experiment. For each donor, mineralization endpoints (Alizarin Red quantification and osteogenic marker expression) were normalized to the matched vector/no‐OM control to account for baseline and inter‐donor variability.

### 2.5 Quantitative Real‐Time Polymerase Chain Reaction (qRT‐PCR) Assay

RNA was extracted using the RNA Purification Kit (ESscience, RN001) according to the manufacturer's instructions. cDNA synthesis was performed using the PrimeScript RT Master Mix kit (Takara, RR036A). qRT‐PCR was conducted using the SYBR Green PCR Kit (Vazyme Biotechnology, Q711) with a StepOnePlus instrument. Relative gene expression was calculated using the 2^‐ΔΔCt method and normalized to β‐actin.

### 2.6 Western Blot Analysis

Human aortic valve tissues or cells were lysed using RIPA buffer (Beyotime Biotechnology, P0013C) containing protease inhibitors for 30 min. Protein concentrations were determined with a BCA Protein Assay Kit. Equal amounts of total protein (40 µg per lane) were separated on 10%–15% SDS‐polyacrylamide gels and transferred to polyvinylidene fluoride membranes (Millipore, ISEQ00010). After blocking, the membranes were incubated with primary antibodies, followed by HRP‐conjugated secondary antibodies. Immunolabeling was detected using an ECL reagent (Millipore, WBULS0500) and visualized with the Tanon 5200 imaging system. For each lane, target protein band intensities were normalized to the corresponding β‐actin signal from the same lane.

### 2.7 Immunofluorescence Staining

Aortic valve tissues and VICs were fixed with 4% paraformaldehyde, permeabilized with 0.2% Triton X‐100, and blocked with 3% BSA. Samples were incubated overnight at 4°C with primary antibodies, followed by incubation with fluorescently conjugated secondary antibodies and counterstaining with DAPI. Imaging was performed using a fluorescence microscope (Leica, Thunder DMi8), and the images were analyzed by ImageJ software.

### 2.8 Transmission Electron Microscopy (TEM)

VICs were fixed in 2.5% glutaraldehyde and subsequently treated with 1% osmium tetroxide and 2% aqueous uranyl acetate. Following graded dehydration, the samples were embedded in resin. Ultrathin sections were prepared using an ultramicrotome and stained with 0.3% lead citrate. Imaging was conducted using an FEI Tecnai G2 Spirit BioTWIN electron microscope (FEI, Hillsboro, OR, USA).

### 2.9 Luciferase Reporter Assay

To characterize the transcriptional regulation of BNIP3, a human BNIP3 promoter fragment spanning −300 to +50 bp relative to the transcription start site (TSS) was cloned into the pGL3‐Basic vector. This specific region was selected based on JASPAR bioinformatic analysis, which identified a high‐scoring predicted KLF5 binding site at −219 to −210 bp (sequence: GCCCCGCCCC). To assess the functional contribution of this site, a mutant reporter construct (Mut) was generated via site‐directed mutagenesis, in which the core binding sequence was altered to GCCCAGAACC. Cells were co‐transfected with the reporter plasmids (WT or Mut) and a Renilla luciferase plasmid (as an internal control) using Lipofectamine 3000 (ThermoFisher Scientific, L3000‐015). Luciferase activity was measured according to the manufacturer's instructions using the Dual‐Luciferase Reporter Assay System (Promega, E1910).

### 2.10 Cell Transfection

Lentivirus‐based constructs, including control short hairpin RNA (sh‐NC), sh‐BNIP3, sh‐KLF5, KLF5 overexpression lentivirus, and its control (Vector), as well as small interfering RNAs targeting p65 (si‐p65) or cGAS (si‐cGAS) and their matched negative controls (si‐NC), were designed and synthesized by Genebay Biotech Co., Ltd. (Nanjing, China). For lentiviral transduction, VICs were treated with lentivirus (MOI = 40) in the presence of polybrene (5 µg/mL) for 12 h, followed by a medium change and recovery in growth medium, as previously described for primary human VICs [35]. In addition, targeting siRNAs or the corresponding scrambled control siRNA (100 nM) were transfected using Lipofectamine 3000 according to the manufacturer's instructions.

### 2.11 Public Transcriptomic, Single‐Cell, Spatial, and Epigenomic Analyses

Public bulk transcriptomic, single‐cell RNA‐seq, spatial transcriptomic, and VIC epigenomic datasets were reanalyzed as described in the Supplemental Methods. Briefly, bulk transcriptomic datasets were analyzed within each cohort to identify reproducibly dysregulated KLF genes. Public human aortic valve scRNA‐seq data were processed using Seurat to annotate major cell types and VIC states and to define the KLF5‐low DAVIC signature. Spatial transcriptomic data were used to project the DAVIC signature and inflammatory/osteogenic module scores onto human valve sections. Public human VIC epigenomic tracks from GSE154513 were used to contextualize the BNIP3 promoter and nominate candidate BNIP3 promoter‐associated contacts. These public epigenomic data were used for regulatory‐context analysis only and were not interpreted as disease‐matched chromatin remodeling or KLF5‐specific distal enhancer binding.

### 2.12 Chromatin Immunoprecipitation (ChIP)

ChIP was performed using the Magna ChIP A/G Chromatin Immunoprecipitation Kit (Sigma‐Aldrich, 17–10086) according to the manufacturer's instructions. Briefly, VICs were cross‐linked, lysed, and sonicated to generate fragmented chromatin. Chromatin was immunoprecipitated using antibodies against KLF5, H3K27ac, H3K4me3, or a normal IgG control, as indicated. After immunoprecipitation, cross‐links were reversed and DNA was purified. KLF5 occupancy was assessed at the BNIP3 promoter region containing the validated KLF5‐responsive motif. Histone ChIP‐qPCR was performed at the BNIP3 promoter P1 and P2 intervals, with P1 used for H3K27ac analysis and P2 used for H3K27ac and H3K4me3 analysis.

### 2.13 Chromosome Conformation Capture‐qPCR (3C‐qPCR)

3C‐qPCR was used to test selected physical proximities between the BNIP3 promoter and public H3K27ac HiChIP‐nominated candidate distal regions. The BNIP3 promoter bait and C1–C3 candidate fragments were defined by in silico HindIII restriction mapping. After the indicated treatments, VICs were cross‐linked with 1% formaldehyde, quenched with glycine, lysed to isolate nuclei, digested with HindIII, and proximity‐ligated under dilute ligation conditions using a Chromosome Conformation Capture Kit according to the manufacturer's instructions. After reverse cross‐linking and DNA purification, 3C products were quantified by qPCR using junction‐specific primer pairs spanning the BNIP3 promoter bait and each candidate fragment. No‐ligation, undigested, no‐template, digestion‐efficiency, and negative‐anchor controls were included. Interaction frequencies were compared within each promoter–candidate pair between sh‐NC and sh‐KLF5 groups.

### 2.14 Mitophagy Analysis

Cells were transduced with GFP‐LC3 lentivirus, and mitochondria were labeled with MitoTracker Red (Beyotime Biotechnology, C1035). Images were acquired using a confocal laser scanning microscope. Colocalization analysis of GFP‐LC3 and MitoTracker Red was performed using ImageJ software. Mitophagic flux was assessed using a mitochondria‐targeted pH‐sensitive mito‐Keima reporter. Signals were acquired sequentially at 440 and 560 nm excitation, and mitophagic flux was quantified as the 560/440 excitation ratio using identical acquisition and analysis settings across groups.

### 2.15 Dihydroethidium (DHE) Staining

Reactive oxygen species (ROS) levels in primary cells or aortic valve tissues were assessed using DHE staining (MedChemExpress, HY‐D0079). DHE was dissolved in dimethyl sulfoxide (DMSO) and diluted to a working concentration of 5 µM. Cells or tissue sections were washed three times with PBS, then incubated with the DHE solution for 30 min. Fluorescence imaging was immediately performed using a microscope (Leica, Thunder DMi8).

### 2.16 Quantification of Mitochondrial DNA Release

VICs or human aortic valve tissues were processed on ice in isotonic buffer containing digitonin (20 µg/mL) and incubated for 10 min at 4°C with gentle rocking. Homogenates were centrifuged at 1,000 × g for 10 min at 4°C to pellet nuclei/debris, and the supernatant was centrifuged at 12,000 × g for 20 min at 4°C to remove mitochondria. DNA was purified from the resulting mitochondrial‐depleted cytosolic fraction using the DNeasy Blood & Tissue Kit (QIAGEN, cat. 69504) and analyzed by qPCR. Cytosolic mtDNA targets (MT‐ND1, MT‐ND2) were measured together with nuclear genomic DNA (L1ORF1, L1ORF2) and ribosomal gene (18S rRNA) markers to verify cytosolic origin. Fractions were accepted only when nuclear/ribosomal markers were ≥6 Ct higher than mtDNA targets; samples failing this QC were excluded. Primer sequences are listed in Table S2.

### 2.17 Seahorse XF Mitochondrial Bioenergetics Assay

Mitochondrial function was evaluated via the Seahorse XF Analyzer per manufacturer specifications. Oxygen consumption rate (OCR) was measured under basal conditions and after sequential injections of oligomycin (1.5 µM), the uncoupler FCCP (2.0 µM), and the complex inhibitors rotenone/antimycin A (0.5 µM). These modulators enabled quantification of basal respiration, ATP‐linked oxygen consumption, maximal respiratory capacity, and spare respiratory capacity. Three consecutive measurements were recorded per compound injection, and mitochondrial parameters were derived using the manufacturer‐defined calculations.

### 2.18 Bioinformatic Analysis

VICs from the KLF5 knockdown groups were lysed in TRIzol reagent (Invitrogen, Carlsbad, CA) for total RNA extraction. cDNA libraries were prepared separately for each biological replicate using the NEBNext Ultra Directional RNA Library Prep Kit (Illumina), following the manufacturer's protocol. RNA‐seq data were processed using the BioJupies platform, including quality control, normalization, and downstream pathway enrichment analysis [36]. Kyoto Encyclopedia of Genes and Genomes (KEGG) enrichment analysis identified biological processes associated with differentially expressed genes, which guided subsequent experimental validation.

### 2.19 Statistical Analysis

Statistical analyses were performed using GraphPad Prism 9.0. Data are presented as mean ± SD unless otherwise stated, and individual data points are shown where applicable. The sample size (n) represents independent biological replicates, including independent patient‐derived valve specimens, independent primary VIC donors or independent cell‐culture experiments, and individual animals. For image‐based analyses, technical measurements from multiple fields, sections, or cells within the same biological specimen, animal, or cell‐culture replicate were averaged to generate one biological replicate‐level value before group‐level statistical testing. All tests were two‐tailed, and *p* < 0.05 was considered statistically significant. Given the modest sample sizes and the potential non‐normal distribution of clinical and molecular variables, correlation analyses were primarily performed using Spearman's rank correlation. Pearson's correlation was used only for prespecified approximately normally distributed variables. Normality was assessed when sample size allowed. For small sample sizes or datasets not meeting normality assumptions, nonparametric tests were used, including the Mann–Whitney U test for two‐group comparisons and the Kruskal–Wallis test followed by Dunn's post hoc test for multiple‐group comparisons. For approximately normally distributed datasets, two‐group comparisons were performed using an unpaired two‐tailed Student's t‐test when appropriate, whereas multiple‐group comparisons were performed using one‐way or two‐way ANOVA with appropriate post hoc multiple‐comparison corrections.

## 3 Results

### 3.1 KLF5 Downregulation in Human CAVD Localizes to Disease‐Associated VIC States

**FIGURE 1:**
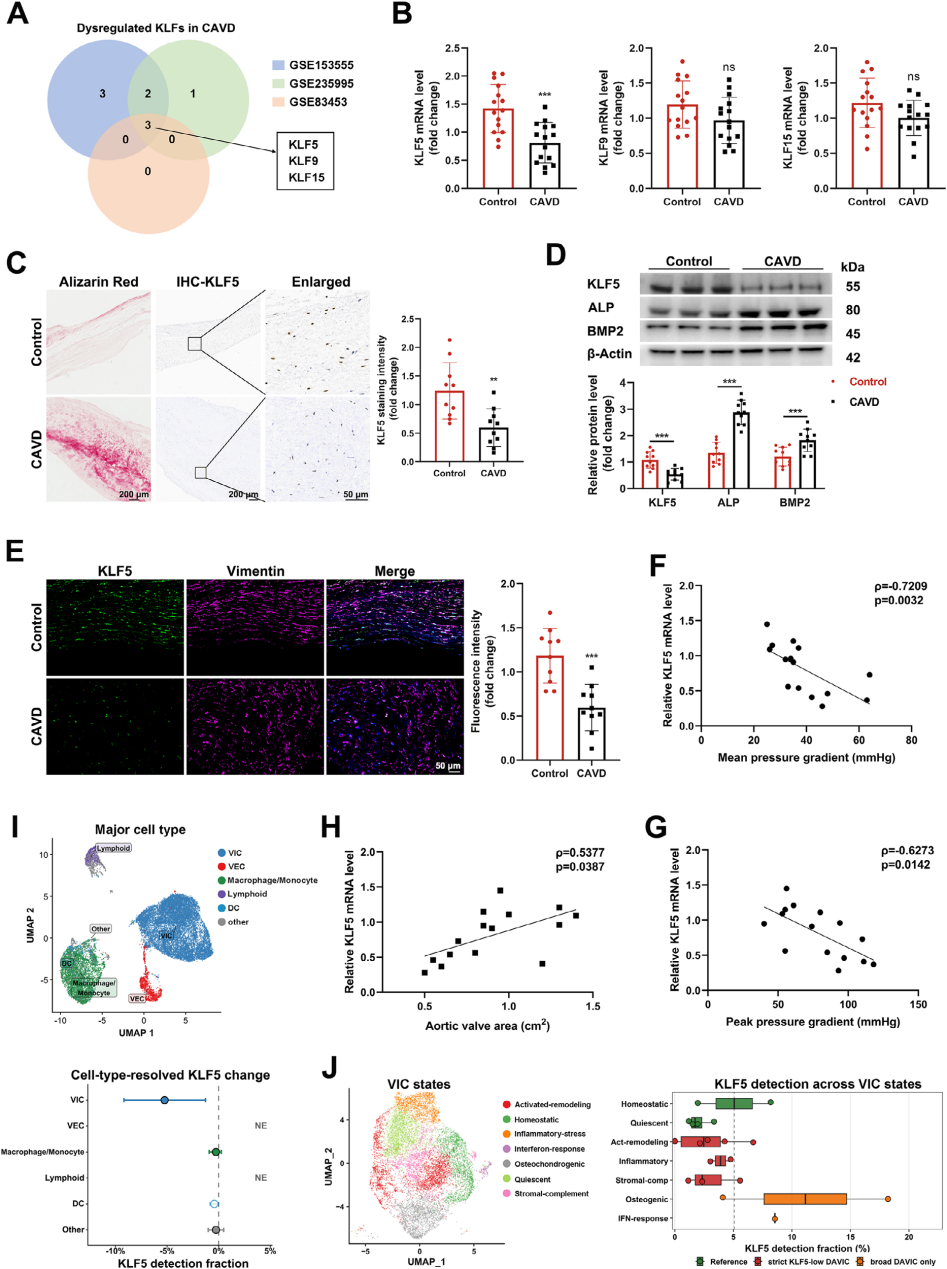
KLF5 downregulation in human CAVD localizes to disease‐associated VIC states. (A) Venn diagram of differentially expressed KLF genes across transcriptomic datasets GSE153555, GSE235995, and GSE83453. (B) KLF5, KLF9, and KLF15 mRNA levels in human aortic valve tissues were measured by qRT‐PCR (n = 15 independent human valve specimens per group). (C) Representative Alizarin Red staining and KLF5 immunohistochemical staining of human aortic valve tissues from non‐calcified reference valves and CAVD valves (n = 10 independent human valve specimens per group; scale bar, 200 µm in overview images and 50 µm in enlarged views of the boxed regions). (D) Western blot analysis of KLF5, BMP2, and ALP protein levels in human aortic valve tissues (n = 10 independent human valve specimens per group). (E) Co‐immunofluorescence staining of KLF5 and Vimentin in human aortic valve tissues from non‐calcified reference valves and CAVD valves, with quantification of KLF5 fluorescence intensity in Vimentin‐positive VIC‐rich regions (n = 10 independent human valve specimens per group; scale bar = 50 µm). (F–H) Spearman correlation analyses between KLF5 mRNA levels and echocardiographic indices of CAVD severity, including mean transvalvular pressure gradient (F), peak transvalvular pressure gradient (G), and aortic valve area (H) in human CAVD specimens (n = 15 independent human valve specimens). Spearman's ρ and *p* values are shown in each plot. (I) Reanalysis of public human aortic valve scRNA‐seq data from GSE291890. Upper: all‐cell UMAP showing major valvular cell compartments, including VICs, VECs, macrophage/monocyte cells, lymphoid cells, dendritic cells, and other minor cell populations. Lower: cell‐type‐resolved AS–AR difference in KLF5 detection fraction, defined as the proportion of cells with KLF5 RNA counts > 0 within each patient/library. Negative values indicate lower KLF5 detection in AS relative to AR. The analysis included two AR patients/two libraries and four AS patients/five libraries, retaining 16,877 AR and 1,688 AS cells after quality‐control filtering. AR samples were used as non‐calcific aortic regurgitation disease comparators rather than healthy controls. (J) UMAP of reclustered VICs annotated into homeostatic, quiescent, activated‐remodeling, inflammatory‐stress, stromal‐complement, osteogenic, and interferon‐response states. KLF5 detection fraction was assessed across VIC states after state annotation using RNA counts > 0. NE indicates not estimable because one comparison group lacked sufficient eligible library–cell‐type coverage. Statistical significance is indicated as ***p < 0.001, **p < 0.01; ns indicates no statistical significance.

To explore the potential role of KLF family members in CAVD, we first analyzed human aortic valve transcriptomic datasets from the GEO database. Using three primary human aortic valve transcriptomic cohorts as a cross‐platform discovery set, we identified KLF5, KLF9, and KLF15 as KLF family members that were differentially expressed across all three within‐cohort disease–reference contrasts (Figure 1A). We then examined these candidates in our human aortic valve cohort, where qRT‐PCR confirmed significant downregulation of KLF5 in CAVD valves compared with non‐calcified reference valves (Figure 1B). The comparator group consisted of non‐calcified tricuspid valves obtained during Bentall procedures or heart transplantation, rather than valves from completely healthy individuals. In contrast, KLF9 and KLF15 did not show significant differences in the validation cohort. Therefore, KLF5 was selected for subsequent validation and mechanistic analysis.

We next examined KLF5 expression in relation to calcified valve lesions using serial histological sections. Alizarin Red staining confirmed prominent calcium deposition in CAVD leaflets, whereas non‐calcified reference valves showed no detectable Alizarin Red‐positive calcium deposition. In anatomically matched sections, KLF5 immunoreactivity was reduced in lesion‐associated regions of CAVD leaflets compared with non‐calcified reference valves (Figure 1C). Consistent with these findings, Western blot analysis demonstrated significantly decreased KLF5 protein levels, along with elevated levels of osteogenic markers ALP and BMP2 in CAVD tissues (Figure 1D). To further determine whether KLF5 reduction involved the valvular interstitial compartment, we performed Vimentin and KLF5 co‐immunofluorescence staining in human aortic valve tissues. KLF5 fluorescence intensity was reduced in Vimentin‐positive VIC‐rich mesenchymal regions of CAVD valves compared with non‐calcified reference valves (Figure 1E). Together, molecular and in situ analyses showed reduced KLF5 expression in CAVD tissues, including lesion‐associated regions and Vimentin‐positive VIC‐rich mesenchymal compartments. To evaluate clinical relevance, KLF5 expression was correlated with echocardiographic parameters. Lower KLF5 mRNA levels were associated with higher mean and peak transvalvular pressure gradients and with smaller aortic valve area in CAVD specimens (Figure 1F–H). These associations were consistent with a relationship between reduced KLF5 expression and hemodynamic severity of CAVD.

Because human aortic valve tissues comprise multiple cellular compartments, we next reanalyzed publicly available human aortic valve scRNA‐seq data from GEO (GSE291890) to resolve KLF5 expression at single‐cell resolution. In this dataset, aortic stenosis (AS) samples were analyzed as the calcified/stenotic disease group, whereas aortic regurgitation (AR) samples were used as non‐calcific disease comparators rather than healthy normal controls. The public human aortic valve scRNA‐seq analysis included six patients represented by seven non‐sorted Flex libraries: two AR patients represented by two libraries and four AS patients represented by five libraries in total, with one AS patient contributing two libraries. After quality‐control filtering, 18,565 cells were retained, including 16,877 AR cells and 1,688 AS cells. UMAP analysis of all cells identified major valvular cell compartments, including VICs, VECs, macrophage/monocyte cells, lymphoid cells, dendritic cells, and other minor populations (Figure 1I). Canonical marker expression supporting these annotations and post‐QC library‐level distributions of detected genes, UMI counts, and mitochondrial transcript percentage are shown in Figure S1A,B. Across the major valve cell populations, KLF5 was preferentially detected in VICs in both groups (Figure S1C). At the library level, KLF5 detection fraction was lower in AS than in AR within VICs. Macrophage/monocyte and Other populations showed AS–AR effect estimates close to zero, whereas VEC and lymphoid comparisons were not estimable because of insufficient eligible library‐level coverage. VICs were then reclustered and annotated according to state‐enriched marker genes before KLF5 detection was assessed, identifying homeostatic, quiescent, activated‐remodeling, inflammatory‐stress, stromal‐complement, osteogenic, and interferon‐response VIC states (Figure 1J). KLF5 detection fraction was quantified across annotated VIC states using RNA counts > 0. Reduced KLF5 detection was observed in activated‐remodeling, inflammatory‐stress, and stromal‐complement VIC states relative to homeostatic VICs. For downstream spatial projection, these remodeling/stress‐associated VIC states were operationally used to define a KLF5‐low disease‐associated VIC (DAVIC) signature. Representative DAVIC signature genes and exploratory gene‐set annotation of KLF5‐low DAVIC signature states relative to homeostatic VICs are provided in Figure S2.

Collectively, these data identify reduced KLF5 expression as a recurrent feature of human CAVD tissues and localize this reduction to Vimentin‐positive VIC‐rich regions and selected KLF5‐low remodeling/stress‐associated VIC states.

### 3.2 KLF5 Modulates OM‐Induced Osteogenic Responses in Primary Human VICs

**FIGURE 2:**
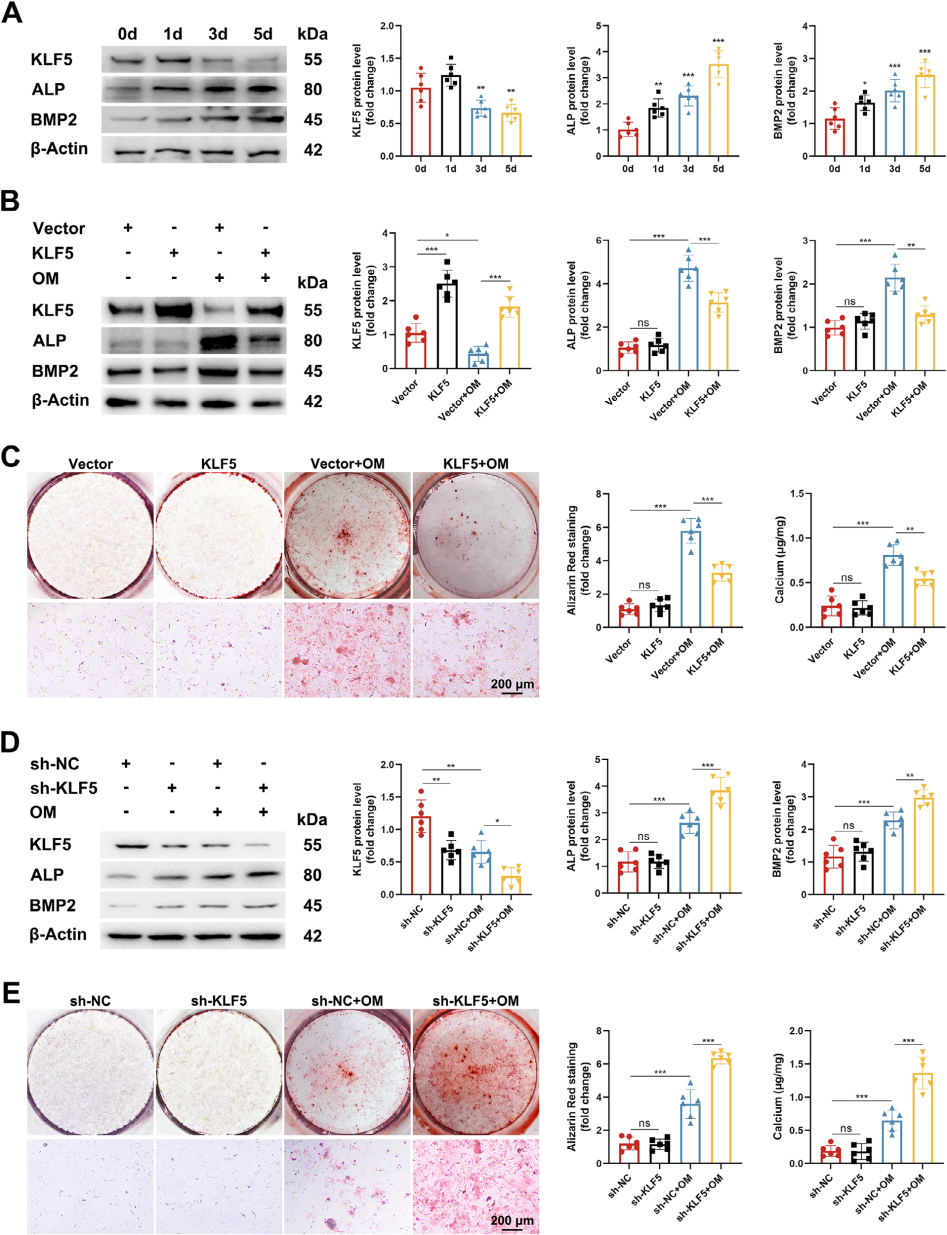
KLF5 modulates OM‐induced osteogenic responses in primary human VICs. (A and B) Western blot analysis of KLF5 and the osteogenic markers BMP2 and ALP in OM‐treated VICs over time (days 0, 1, 3, and 5) (A) and in KLF5‐overexpressing VICs under OM conditions (B) (n = 6 independent biological replicates per group). (C) Alizarin Red staining of mineralized nodules revealed calcium deposition in the indicated groups (n = 6 independent biological replicates per group; scale bar = 200 µm). (D) Western blot analysis of BMP2 and ALP protein expression in sh‐KLF5 treated VICs (n = 6 independent biological replicates per group). (E) Alizarin Red staining was used to assess calcium deposition in VICs (n = 6 independent biological replicates per group; scale bar = 200 µm). Statistical significance is indicated as ***p < 0.001, **p < 0.01, *p < 0.05, ns indicates no statistical significance.

Following the identification of reduced KLF5 expression in human CAVD tissues and selected disease‐associated VIC states, we examined whether KLF5 altered the response of primary human VICs to pro‐osteogenic stimulation. To minimize potential confounding from the spontaneous calcification reported in VICs derived from CAVD valves [37], all in vitro osteogenic assays were performed using primary human VICs isolated from non‐calcified valves. Under osteogenic medium, KLF5 expression decreased in a time‐dependent manner, whereas the osteogenic markers ALP and BMP2 were progressively upregulated (Figure 2A). These observations indicated that KLF5 reduction accompanies the OM‐induced osteogenic response of VICs; therefore, gain‐ and loss‐of‐function analyses were performed to assess the effect of KLF5 on this response.

To determine whether maintaining KLF5 expression influences OM‐induced osteogenic responses, VICs were infected with KLF5 overexpression lentivirus or the corresponding control (Vector) and then exposed to OM. Compared with Vector controls, KLF5 overexpression significantly reduced OM‐induced ALP and BMP2 upregulation (Figure 2B). Furthermore, VICs overexpressing KLF5 exhibited reduced matrix mineralization and calcium deposition during osteogenic differentiation compared with the control group (Figure 2C).

Conversely, VICs were infected with sh‐KLF5 or the control sh‐NC prior to OM treatment. KLF5 knockdown augmented the OM‐induced osteogenic response, as evidenced by increased ALP and BMP2 expression (Figure 2D) and increased calcium deposition and nodule formation relative to sh‐NC controls (Figure 2E). Notably, neither KLF5 overexpression nor knockdown produced appreciable mineral deposition in the absence of osteogenic medium, indicating that KLF5 does not initiate spontaneous mineralization under basal culture conditions but modulates the magnitude of the OM‐induced mineralizing response.

Together, these data indicate that KLF5 modulates the magnitude of OM‐induced osteogenic and mineralizing responses in primary human VICs under inductive culture conditions.

### 3.3 KLF5 Overexpression Attenuates HFD‐associated Aortic Valve Remodeling in ApoE^−/−^ Mice

To evaluate whether KLF5 overexpression altered aortic valve remodeling in vivo, ApoE^−/−^ mice were assigned to normal diet or high‐fat diet conditions and received tail‐vein injection of AAV‐KLF5 or the corresponding AAV‐scr control vector. Assessment after AAV‐KLF5 administration showed increased KLF5 signal in aortic valve leaflets, including within Vimentin‐positive regions (Figure S3A,B). Extra‐valvular assessment showed increased hepatic KLF5 expression, whereas renal KLF5 expression was not significantly altered (Figure S4A–C). Serum lipid profiles, cardiac functional indices, and hepatic and renal biochemical parameters were comparable between HFD+AAV‐scr and HFD+AAV‐KLF5 mice (Table S3).

**FIGURE 3:**
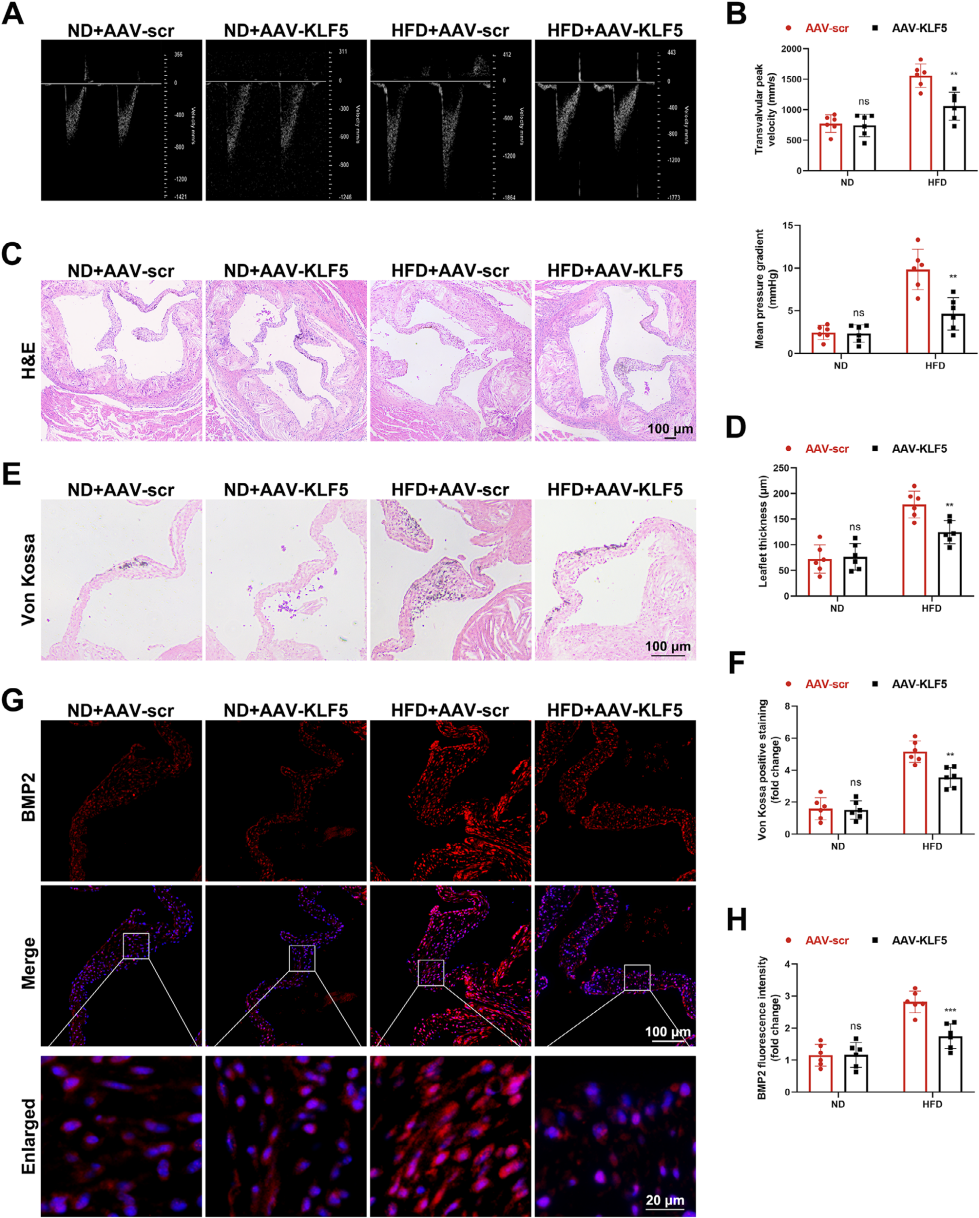
KLF5 overexpression attenuates HFD‐associated aortic valve remodeling in ApoE^−/−^ mice. (A and B) Peak transvalvular jet velocity and mean transvalvular pressure gradient were measured by echocardiography (n = 6 individual mice per group). (C and D) Representative H&E staining and quantification of aortic valve leaflet thickness in ApoE^−/−^ mice injected via tail vein with AAV‐scr or AAV‐KLF5 (n = 6 individual mice per group; scale bar = 100 µm). (E and F) Calcium deposition was observed by Von Kossa staining in the indicated groups (n = 6 individual mice per group; scale bar = 100 µm). (G and H) Representative immunofluorescence staining images of BMP2 in aortic valve leaflets of mice (n = 6 individual mice per group; scale bar, 100 µm in overview images and 20 µm in enlarged views of the boxed regions). Statistical significance is indicated as ***p < 0.001, **p < 0.01, ns indicates no statistical significance.

After 20 weeks, HFD+AAV‐scr mice exhibited significant increases in aortic valve flow velocity and transvalvular pressure gradients compared with ND+AAV‐scr mice, consistent with HFD‐associated valvular hemodynamic dysfunction (Figure 3A,B). Compared with HFD+AAV‐scr mice, HFD+AAV‐KLF5 mice showed lower aortic valve flow velocity and transvalvular pressure gradients. Histological analysis, including H&E and Von Kossa staining, revealed increased leaflet thickness and calcium deposition in HFD+AAV‐scr mice, whereas both measurements were lower in HFD+AAV‐KLF5 mice (Figure 3C–F). Immunofluorescence analysis showed increased BMP2 signal in valve leaflets from HFD+AAV‐scr mice and lower BMP2 signal in HFD+AAV‐KLF5 mice (Figure 3G,H).

In summary, these in vivo findings indicate that AAV2‐mediated KLF5 overexpression attenuated HFD‐associated valvular hemodynamic abnormalities, leaflet thickening, mineral deposition, and BMP2 expression in ApoE^−/−^ mice.

### 3.4 KLF5 Loss Promotes Cytosolic mtDNA Accumulation and cGAS–STING Activation

**FIGURE 4:**
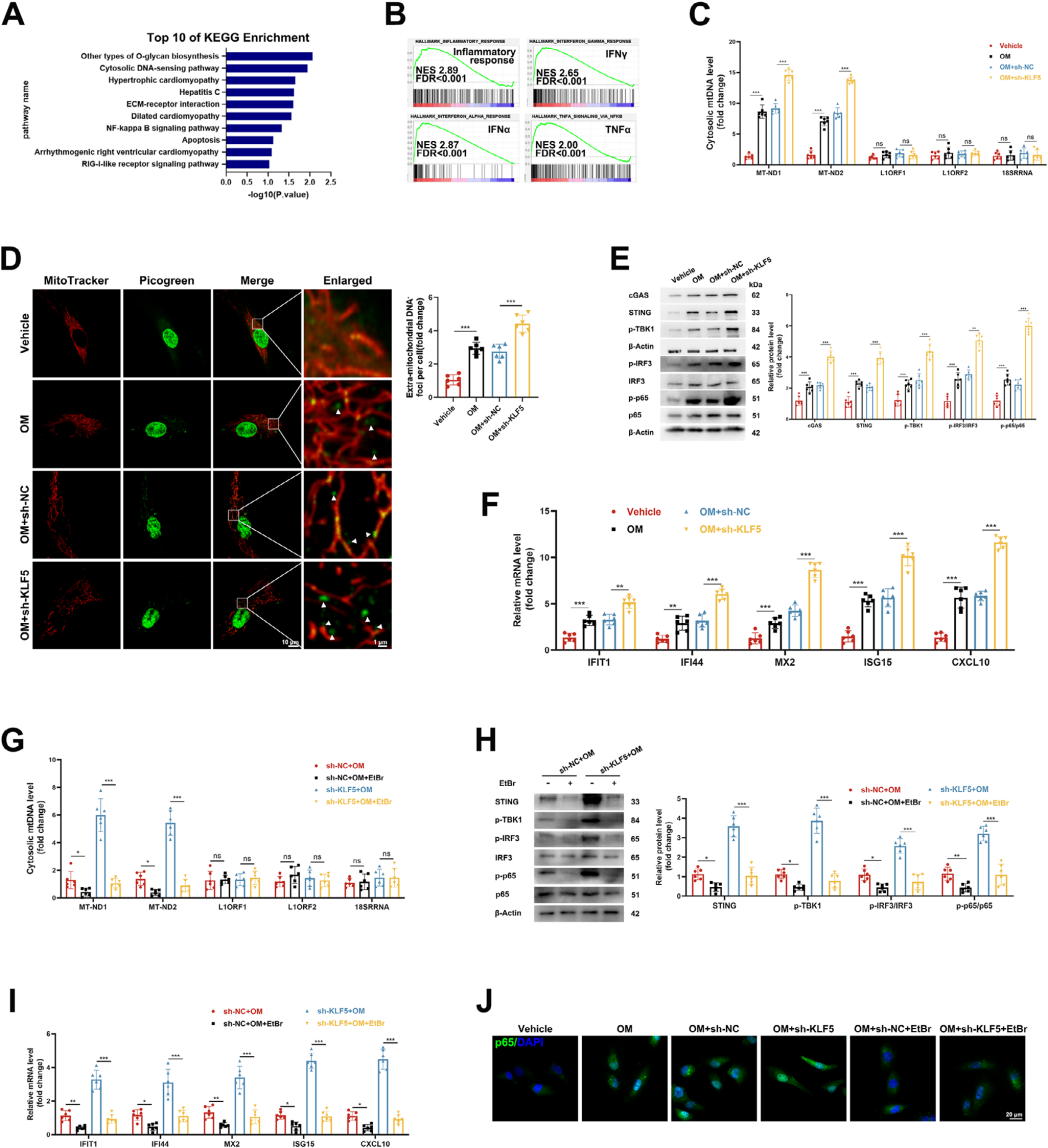
KLF5 loss promotes cytosolic mtDNA accumulation and cGAS–STING activation. (A and B) KEGG enrichment analysis and GSEA of RNA‐seq data from KLF5‐deficient VICs, showing enrichment of cytosolic DNA‐sensing, interferon signaling, TNF‐α signaling, and inflammatory response pathways. (C) qPCR measurement of cytosolic mtDNA (specifically MT‐ND1 and MT‐ND2), nuclear LINE1 elements, and 18S rRNA levels (n = 6 independent biological replicates per group). (D) Confocal microscopy showing extra‐mitochondrial DNA^+^ foci in the cytoplasm of OM‐treated VICs (arrowheads indicate cytosolic DNA outside mitochondria). Extra‐mitochondrial DNA was quantified as PicoGreen signal not overlapping with MitoTracker (n = 6 independent biological replicates per group; scale bar, 10 µm in overview images and 1 µm in enlarged views of the boxed regions). (E) Western blot analysis of cGAS, STING, p‐TBK1, IRF3, p‐IRF3, p65, and p‐p65 protein levels (n = 6 independent biological replicates per group). (F) qRT‐PCR measurement of ISGs (IFIT1, IFI44, MX2, ISG15, and CXCL10) mRNA levels (n = 6 independent biological replicates per group). (G) The MT‐ND1, MT‐ND2, nuclear LINE1 elements, and 18S rRNA were measured in OM and sh‐KLF5 groups treated with EtBr (n = 6 independent biological replicates per group). (H) Western blot analysis of STING, p‐TBK1, IRF3, p‐IRF3, p65, and p‐p65 protein levels in the different groups (n = 6 independent biological replicates per group). (I) ISG mRNA levels were measured in the OM and sh‐KLF5 groups treated with EtBr (n = 6 independent biological replicates per group). (J) Immunofluorescence staining of p65 in the indicated groups (n = 5 independent biological replicates per group; scale bar = 20 µm). Statistical significance is indicated as ***p < 0.001, **p < 0.01, *p < 0.05, ns indicates no statistical significance.

Following the observation that KLF5 modulates OM‐induced osteogenic and mineralizing responses in primary human VICs, we next examined the signaling pathways associated with KLF5 loss. RNA sequencing of KLF5‐deficient VICs showed enrichment of cytosolic DNA‐sensing and NF‐κB‐related pathways (Figure 4A). GSEA further identified interferon signaling, TNF‐α signaling, and inflammatory response signatures (Figure 4B). These transcriptomic findings identified DNA‐sensing and inflammatory signaling pathways for experimental validation.

Because cytosolic DNA may arise from multiple sources, including nuclear and mitochondrial leakage, we next examined its potential source [38]. qPCR analysis with source‐specific primers revealed significant increases in cytosolic mtDNA (MT‐ND1, MT‐ND2) following OM treatment, which were further elevated upon KLF5 knockdown (Figure 4C). In contrast, the nuclear DNA markers (L1ORF1, L1ORF2) and ribosomal gene (18S rRNA) did not show comparable changes under these conditions. Consistently, MitoTracker and PicoGreen co‐staining detected extranuclear DNA accumulation in the cytoplasm outside mitochondria in OM‐treated VICs, with this effect appearing more pronounced upon KLF5 knockdown (Figure 4D). These observations indicate that OM‐induced VIC remodeling is accompanied by cytosolic mtDNA accumulation and that KLF5 knockdown further enhances this response.

Given that cytosolic mtDNA can activate the cGAS–STING pathway, we next assessed this axis [21]. Western blot analysis showed OM‐induced upregulation of cGAS, STING, p‐TBK1, p‐IRF3, and p‐p65, all of which were potentiated by KLF5 knockdown (Figure 4E). In parallel, qRT‐PCR revealed increased expression of interferon‐stimulated genes (ISGs), including IFIT1, IFI44, MX2, ISG15, and CXCL10, an effect that was further augmented upon KLF5 depletion (Figure 4F).

To directly assess whether the DNA‐sensing response enhanced by KLF5 deficiency depended on cGAS, we performed siRNA‐mediated cGAS knockdown in OM‐treated VICs. cGAS knockdown reduced cGAS expression and attenuated the increases in STING, p‐TBK1, p‐IRF3/IRF3, and p‐p65/p65 observed in KLF5‐deficient VICs (Figure S5A). Consistently, si‐cGAS reduced the induction of IFIT1, IFI44, MX2, ISG15, and CXCL10 under KLF5‐deficient conditions (Figure S5B). These data indicate that the amplified inflammatory response was partly dependent on cGAS.

To test whether mtDNA contributed to cGAS–STING pathway activation under OM and KLF5‐deficient conditions, mtDNA was depleted using EtBr, with depletion confirmed by reduced mtDNA levels (Figure 4G). EtBr‐mediated mtDNA depletion attenuated cGAS–STING pathway activation in OM‐treated and KLF5‐deficient VICs and reduced the induction of the aforementioned ISGs (Figure 4H,I). Furthermore, immunofluorescence analysis showed that OM stimulation increased both cytosolic and nuclear p65 levels, with nuclear accumulation further enhanced by KLF5 knockdown (Figure 4J). EtBr treatment reduced p65 signal intensity and nuclear translocation under these conditions. These results indicate that cytosolic mtDNA contributes to cGAS–STING and NF‐κB signaling activation in OM‐treated, KLF5‐deficient VICs.

Consistent with the cell‐based findings, interferon‐ and inflammation‐related Hallmark signatures were also enriched in an independent public human aortic valve transcriptomic cohort (GSE153555; Figure S6A). Moreover, human calcified aortic valve tissues showed increased cytosolic mtDNA levels, enhanced cGAS–STING pathway activation, and elevated ISG transcripts compared with non‐calcified reference valves (Figure S6B–D). Together, these data indicate that KLF5 deficiency enhances OM‐associated cytosolic mtDNA accumulation and cGAS–STING/NF‐κB signaling in primary human VICs, while human CAVD tissues show concordant activation of this DNA‐sensing inflammatory axis.

### 3.5 KLF5 Loss Enhances mtDNA‐Sensitive STING/NF‐κB Signaling and NLRP3‐Associated Inflammatory Responses in VICs

The mtDNA–cGAS–STING/NF‐κB pathway activation identified above prompted us to examine whether this inflammatory signaling program was associated with NLRP3‐related inflammatory output in VICs. Because NF‐κB can transcriptionally regulate NLRP3 [39, 40], we next examined NLRP3‐associated inflammatory responses under OM and KLF5‐deficient conditions. OM treatment increased NLRP3, cleaved caspase‐1, and IL‐1β protein levels in VICs (Figure S7A). In parallel, qRT‐PCR showed increased IL‐1β and TNF‐α transcripts, and ELISA confirmed elevated secretion of these cytokines into the culture supernatant (Figure S7B,C). TNF‐α was analyzed as an NF‐κB–responsive inflammatory cytokine output, whereas inflammasome‐associated signaling was assessed by NLRP3, cleaved caspase‐1, and IL‐1β.

**FIGURE 5:**
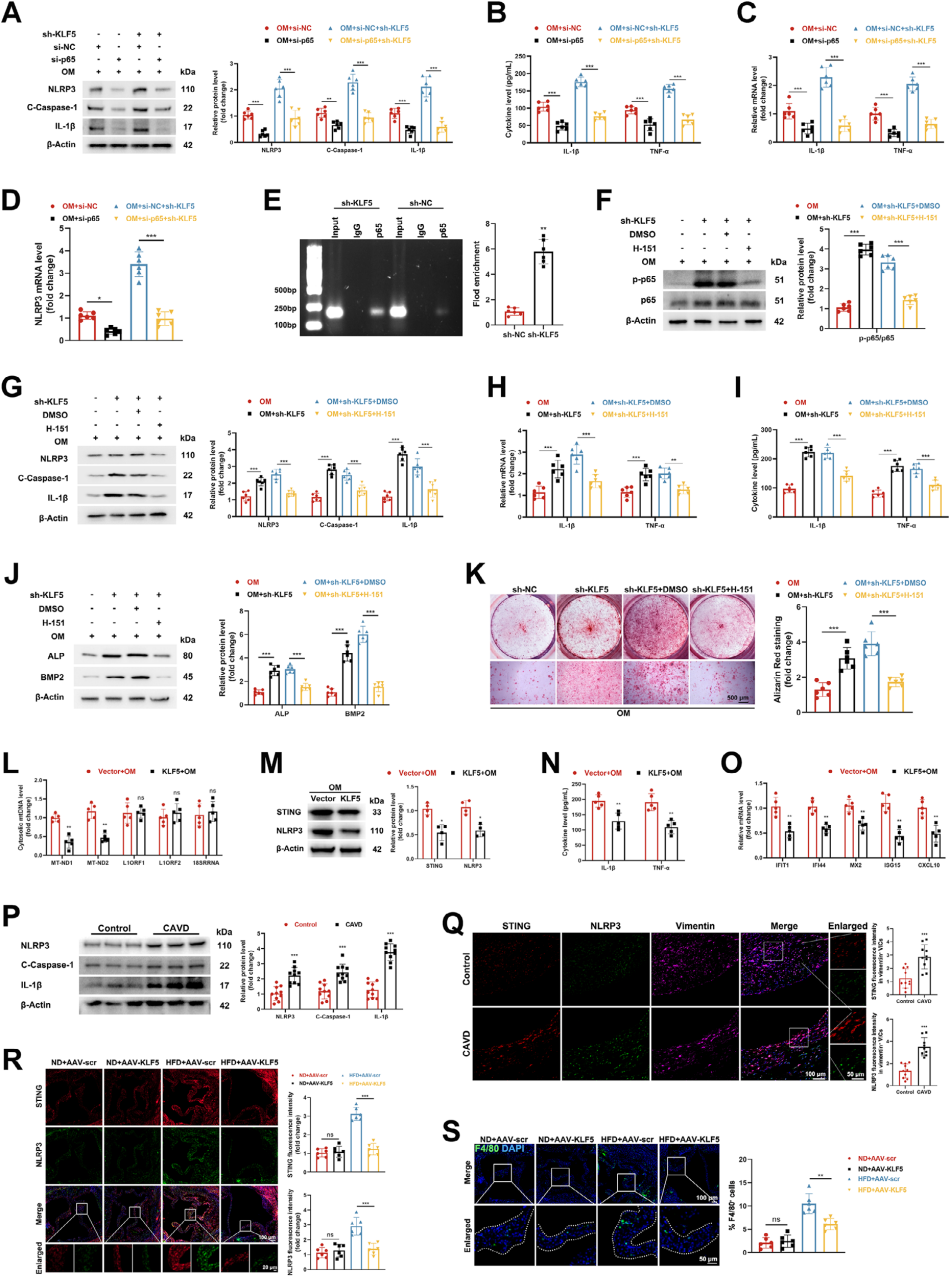
KLF5 loss enhances mtDNA‐sensitive STING/NF‐κB signaling and NLRP3‐associated inflammatory responses in VICs. (A) Western blot analysis of NLRP3, cleaved caspase‐1, and IL‐1β protein levels in KLF5‐deficient VICs with si‐p65 (n = 6 independent biological replicates per group). (B) ELISA measurements of IL‐1β and TNF‐α concentrations in cell supernatants (n = 6 independent biological replicates per group). (C) qRT‐PCR measurement of IL‐1β and TNF‐α mRNA levels (n = 6 independent biological replicates per group). (D) NLRP3 mRNA levels were detected in the indicated groups (n = 6 independent biological replicates per group). (E) ChIP assay demonstrated increased p65 binding to the NLRP3 promoter in KLF5‐deficient VICs (n = 6 independent biological replicates per group). (F) Western blot analysis of p‐p65 protein levels in VICs treated with STING inhibitor H‐151 (n = 6 independent biological replicates per group). (G) NLRP3, cleaved caspase‐1, and IL‐1β protein levels were measured in different groups (n = 6 independent biological replicates per group). (H) IL‐1β and TNF‐α mRNA levels in KLF5‐deficient VICs treated with H‐151 (n = 6 independent biological replicates per group). (I) ELISA measurements of IL‐1β and TNF‐α concentrations in the indicated groups (n = 6 independent biological replicates per group). (J) Western blot analysis of osteogenic markers BMP2 and ALP protein levels (n = 6 independent biological replicates per group). (K) Alizarin Red staining of mineralized nodules revealed calcium deposition in VICs (n = 6 independent biological replicates per group; scale bar = 500 µm). (L‐O) Reciprocal analysis in KLF5‐overexpressing VICs under OM conditions, including qPCR measurement of cytosolic mtDNA targets, nuclear LINE1 elements, and 18S rRNA (L; n = 5 independent biological replicates per group), Western blot analysis of STING and NLRP3 protein levels (M; n = 4 independent biological replicates using primary human VICs per group), ELISA measurement of IL‐1β and TNF‐α concentrations in cell supernatants (N; n = 5 independent biological replicates using primary human VICs per group), and qRT‐PCR measurement of ISG transcripts, including IFIT1, IFI44, MX2, ISG15, and CXCL10 (O; n = 5 independent biological replicates per group). (P and Q) Human valve tissue validation showing NLRP3, cleaved caspase‐1, and IL‐1β protein levels by Western blot analysis (P), and immunofluorescence staining of STING and NLRP3 in Vimentin‐positive VIC‐rich regions of human aortic valve tissues (Q) (n = 10 independent human valve specimens per group; scale bar, 100 µm in overview images and 50 µm in enlarged views of the boxed regions for panel Q). (R and S) Mouse valve tissue validation showing immunofluorescence staining of STING and NLRP3 in aortic valve leaflets from HFD‐fed mice treated with AAV‐scr or AAV‐KLF5 (R), and quantification of F4/80‐positive cells as a readout of the inflammatory valve microenvironment (S) (n = 6 individual mice per group; for panel R, scale bars are 100 µm in overview images and 20 µm in enlarged views; for panel S, scale bars are 100 µm in overview images and 50 µm in enlarged views). Statistical significance is indicated as ***p < 0.001, **p < 0.01, *p < 0.05, and ns indicates no statistical significance.

To assess the contribution of NF‐κB signaling, p65 expression was inhibited using small interfering RNA in KLF5‐deficient VICs under OM conditions. p65 knockdown attenuated the induction of NLRP3, cleaved caspase‐1, and IL‐1β in KLF5‐deficient VICs (Figure 5A). Consistently, ELISA confirmed reduced secretion of IL‐1β and TNF‐α (Figure 5B), and qRT‐PCR showed reduced expression of NLRP3, IL‐1β, and TNF‐α transcripts (Figure 5C,D). Furthermore, ChIP assays revealed increased p65 occupancy at the NLRP3 promoter upon KLF5 knockdown (Figure 5E).

To pharmacologically assess the involvement of STING activity in this inflammatory pathway, we treated KLF5‐deficient VICs with the STING inhibitor H‐151. H‐151 attenuated p65 phosphorylation and reduced the induction of NLRP3, cleaved caspase‐1, and IL‐1β caused by KLF5 knockdown (Figure 5F,G). Similarly, qPCR and ELISA showed that H‐151 reduced IL‐1β and TNF‐α expression and release (Figure 5H,I). Under the same conditions, H‐151 also reduced ALP and BMP2 expression and decreased calcium deposition in KLF5‐deficient VICs (Figure 5J,K). These data indicate that pharmacological STING inhibition attenuated NF‐κB/NLRP3‐associated inflammatory signaling and OM‐induced osteogenic‐marker expression and matrix mineralization under KLF5‐deficient conditions.

As a reciprocal gain‐of‐function analysis, we assessed cytosolic mtDNA accumulation and STING/NLRP3‐associated inflammatory signaling in KLF5‐overexpressing VICs under OM treatment. KLF5 overexpression reduced cytosolic mtDNA accumulation, including MT‐ND1 and MT‐ND2 levels, with no comparable changes in nuclear DNA markers or 18S rRNA (Figure 5L). KLF5 overexpression also reduced STING and NLRP3 protein expression (Figure 5M), decreased IL‐1β and TNF‐α secretion (Figure 5N), and suppressed ISG induction, including IFIT1, IFI44, MX2, ISG15, and CXCL10 (Figure 5O). These reciprocal gain‐of‐function findings indicate that KLF5 limits cytosolic mtDNA accumulation and downstream STING/NLRP3‐associated inflammatory signaling in OM‐treated VICs.

Human valve tissue analyses showed corresponding changes in this inflammatory axis in situ. Compared with non‐calcified reference valves, CAVD valves showed increased NLRP3, cleaved caspase‐1, and IL‐1β protein levels (Figure 5P). Co‐immunofluorescence staining showed increased STING and NLRP3 signals within Vimentin‐positive VIC‐rich mesenchymal regions of diseased valves (Figure 5Q). In the HFD‐associated mouse model, aortic valve leaflets from AAV‐KLF5‐treated mice showed lower STING and NLRP3 signals than those from AAV‐scr‐treated mice (Figure 5R). F4/80‐positive cell signal was also lower in AAV‐KLF5‐treated mice, consistent with a less inflammatory valve leaflet microenvironment (Figure 5S).

Together, these findings show that KLF5 loss is associated with enhanced mtDNA‐sensitive STING/NF‐κB pathway activation and increased NLRP3‐associated inflammatory output in OM‐treated VICs. Pharmacological STING inhibition attenuated both inflammatory cytokine production and OM‐induced mineralizing responses under KLF5‐deficient conditions. KLF5 gain‐of‐function, human valve tissue, and mouse valve data provided concordant cellular and tissue‐level support for activation of this inflammatory axis during calcific valve remodeling.

### 3.6 KLF5 Transcriptionally Activates BNIP3 to Promote Mitophagy and Limit Inflammatory Signaling and Mineralization

**FIGURE 6:**
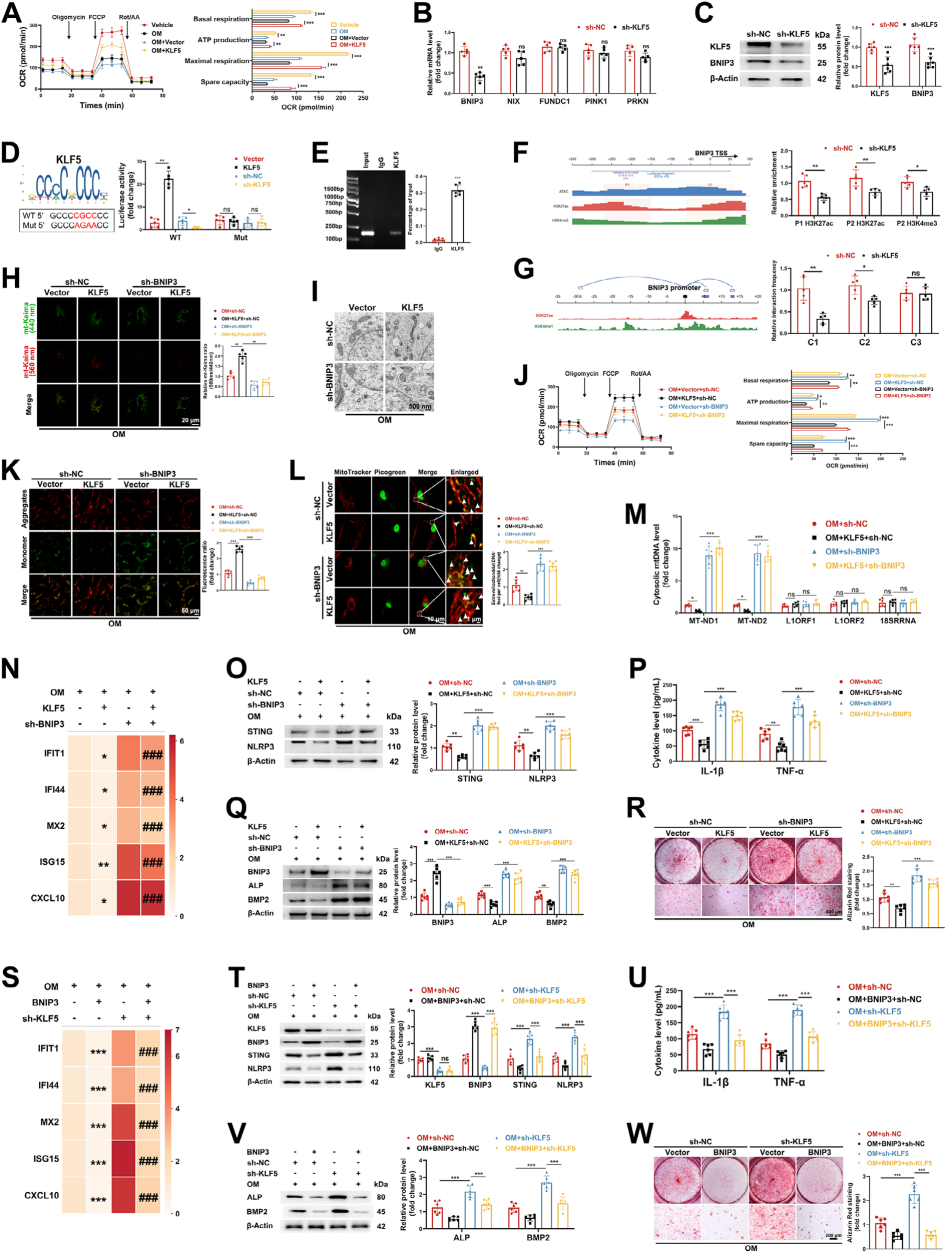
KLF5 transcriptionally activates BNIP3 to promote mitophagy and limit inflammatory signaling and mineralization. (A) The OCR was determined by Seahorse experiments in KLF5‐overexpressing groups (n = 5 independent biological replicates per group). (B) qRT‐PCR analysis of mitophagy‐related genes (BNIP3, NIX, FUNDC1, PINK1, and PRKN) in the KLF5‐knockdown VICs (n = 5 independent biological replicates per group). (C) Western blot analysis of KLF5 and BNIP3 protein levels in KLF5‐deficient VICs (n = 6 independent biological replicates per group). (D) Luciferase reporter assay showing BNIP3 promoter activity (wild‐type or mutant) in KLF5‐overexpressing or knockdown VICs (n = 5 independent biological replicates per group). (E) ChIP assay showing KLF5 binding to the BNIP3 promoter, as detected by qPCR (n = 6 independent biological replicates per group). (F) Public human VIC ATAC, H3K27ac, and H3K4me3 tracks at the BNIP3 promoter, with the luciferase‐tested promoter fragment, validated KLF5‐responsive motif, and P1/P2 ChIP‐qPCR target intervals indicated (n = 5 applies to the accompanying ChIP‐qPCR analysis). (G) Public H3K27ac HiChIP‐nominated BNIP3 promoter‐associated candidate contacts and 3C‐qPCR validation. C1–C3 indicate candidate distal contact regions shown with H3K27ac and H3K4me1 tracks (n = 5 applies to the accompanying 3C‐qPCR analysis). (H) Mito‐Keima‐based assessment of mitophagic flux in KLF5‐overexpressing VICs with or without BNIP3 knockdown under OM treatment (n = 5 independent biological replicates per group; scale bar = 20 µm). (I) Representative TEM images showing mitochondrial ultrastructure and autophagic structures containing mitochondrial profiles in the indicated groups (n = 6 independent biological replicates per group; scale bar = 500 nm). (J) Seahorse XF analysis of OCR in KLF5‐overexpressing VICs with or without BNIP3 knockdown under OM treatment (n = 5 independent biological replicates per group). (K) JC‐1 staining showing mitochondrial membrane potential in KLF5‐overexpressing VICs with or without BNIP3 knockdown (n = 5 independent biological replicates per group; scale bar = 50 µm). (L) Confocal microscopy showing extra‐mitochondrial DNA‐positive foci by MitoTracker and PicoGreen co‐staining in the indicated groups. Arrowheads indicate cytosolic DNA signals outside mitochondria. Quantification of extra‐mitochondrial DNA‐positive foci is shown (n = 6 independent biological replicates per group; scale bar, 10 µm in overview images and 1 µm in enlarged views of the boxed regions). (M) The MT‐ND1, MT‐ND2, nuclear LINE1 elements, and 18S rRNA were measured in KLF5‐overexpressing VICs treated with BNIP3 knockdown (n = 6 independent biological replicates per group). (N‐R) Effects of BNIP3 knockdown on inflammatory signaling and mineralization in KLF5‐overexpressing VICs under OM treatment, including heatmap visualization of ISG transcript levels (N), Western blot analysis of STING and NLRP3 protein levels (O), ELISA measurement of IL‐1β and TNF‐α concentrations in cell culture supernatants (P), Western blot analysis of BNIP3, ALP, and BMP2 protein levels (Q), and Alizarin Red staining showing calcium deposition (R) (n = 6 independent biological replicates per group; scale bar = 500 µm). (S‐W) Effects of BNIP3 restoration in KLF5‐deficient VICs under OM treatment, including heatmap visualization of ISG transcript levels (S), Western blot analysis of KLF5, BNIP3, STING, and NLRP3 protein levels (T), ELISA measurement of IL‐1β and TNF‐α concentrations in cell culture supernatants (U), Western blot analysis of ALP and BMP2 protein levels (V), and Alizarin Red staining showing calcium deposition (W) (n = 6 independent biological replicates per group; scale bar = 200 µm). Statistical significance is indicated as ***p < 0.001, **p < 0.01, *p < 0.05, and ###*p* < 0.001 for the indicated comparisons; ns indicates no statistical significance.

Given that sustained cGAS–STING activation can be driven by cytosolic mtDNA released from damaged mitochondria [15, 21], we investigated whether KLF5 influences mitochondrial integrity. Seahorse XF analysis showed that OM impaired mitochondrial bioenergetic parameters, including basal respiration, ATP‐linked respiration, maximal respiration, and spare respiratory capacity, whereas KLF5 overexpression partially restored these parameters under OM conditions (Figure 6A). Additional mitochondrial quality‐control assays showed that KLF5 overexpression attenuated OM‐induced mitochondrial depolarization, reduced ROS accumulation, improved mitochondrial ultrastructure, and increased GFP‐LC3/MitoTracker colocalization (Figure S8A–D). Because mitophagy is involved in the clearance of damaged mitochondria, we further used pharmacological perturbation to examine its relationship with cytosolic mtDNA accumulation. Pharmacological autophagy inhibition with 3‐MA increased cytosolic mtDNA levels in KLF5‐overexpressing VICs, whereas apoptosis inhibition with Z‐VAD‐FMK did not reduce cytosolic mtDNA accumulation in KLF5‐deficient VICs (Figure S8E). Together, these observations indicate that KLF5 helps preserve mitochondrial quality control and limits cytosolic mtDNA accumulation under OM conditions.

To define the molecular basis of this regulation, we screened mitophagy‐related genes after KLF5 knockdown. Among BNIP3, NIX, FUNDC1, PINK1, and PRKN, BNIP3 showed the most prominent reduction after KLF5 depletion (Figure 6B). Western blot analysis confirmed that KLF5 knockdown decreased BNIP3 protein abundance (Figure 6C). Bioinformatic analysis identified a predicted KLF5 binding motif in the BNIP3 promoter. Luciferase reporter assays showed that KLF5 overexpression increased BNIP3 promoter activity, whereas KLF5 knockdown reduced it; mutation of the predicted KLF5 binding motif abolished these promoter responses (Figure 6D). ChIP assays further showed KLF5 enrichment at the BNIP3 promoter (Figure 6E). These data identify BNIP3 as a transcriptional target of KLF5 in VICs.

To place this experimentally validated KLF5–BNIP3 promoter interaction within a human VIC chromatin context, we analyzed public human VIC ATAC‐seq and histone ChIP‐seq tracks from GSE154513. The BNIP3 promoter region encompassing the luciferase‐tested promoter fragment and the validated KLF5‐responsive motif was marked by accessible chromatin and active promoter‐associated H3K27ac and H3K4me3 signals (Figure 6F). Guided by these regulatory tracks, we designed ChIP‐qPCR assays at a motif‐proximal promoter interval (P1) and a TSS‐proximal promoter interval (P2). KLF5 knockdown reduced H3K27ac enrichment at both P1 and P2 and decreased H3K4me3 enrichment at P2, indicating attenuation of active promoter‐associated chromatin marks at the BNIP3 promoter after KLF5 depletion. Public H3K27ac HiChIP data further nominated candidate BNIP3 promoter‐associated contacts with nearby H3K27ac/H3K4me1‐marked regions. We prioritized candidate fragments C1–C3 for targeted Chromatin conformation capture assay‐qPCR (3C‐qPCR) validation. 3C‐qPCR showed that promoter–C1 and promoter–C2 interaction frequencies were reduced after KLF5 knockdown, whereas the promoter–C3 contact was not significantly altered (Figure 6G). Together with the promoter reporter and KLF5 ChIP results, the chromatin data indicate promoter‐level regulation of BNIP3 by KLF5 and show that KLF5 depletion is accompanied by reduced active promoter chromatin and selected promoter‐associated contact frequencies at the BNIP3 locus.

We next examined whether BNIP3 functionally mediated the mitochondrial effects of KLF5 under OM conditions. To directly assess mitophagic flux, we used a mitochondria‐targeted pH‐sensitive mito‐Keima reporter. KLF5 overexpression increased the acidic/basic mito‐Keima ratio in OM‐treated VICs, consistent with enhanced delivery of mitochondria to acidic lysosomal compartments. This increase was markedly attenuated by BNIP3 knockdown (Figure 6H). BNIP3 knockdown also reduced the preservation of mitochondrial ultrastructure and the presence of autophagic structures containing mitochondria (Figure 6I), impaired mitochondrial respiration (Figure 6J), and reduced mitochondrial membrane potential (Figure 6K). BNIP3 knockdown also increased extra‐mitochondrial PicoGreen‐positive DNA foci (Figure 6L) and elevated cytosolic MT‐ND1 and MT‐ND2 abundance despite KLF5 overexpression, whereas nuclear DNA markers and 18S rRNA were not comparably increased (Figure 6M). Additional DHE staining showed that BNIP3 knockdown blunted the ROS‐lowering effect of KLF5 under OM conditions (Figure S9A). These data identify BNIP3 as an important mediator of KLF5‐associated mitophagic flux, mitochondrial preservation, and the limitation of cytosolic mtDNA accumulation under OM conditions.

We then examined whether BNIP3 loss attenuated the suppression of inflammatory signaling by KLF5 overexpression. In KLF5‐overexpressing VICs, BNIP3 knockdown increased ISG expression, STING and NLRP3 protein abundance, and IL‐1β and TNF‐α secretion under OM conditions (Figure 6N–P). Consistently, p65 immunostaining showed increased nuclear accumulation after BNIP3 knockdown (Figure S9B). These findings indicate that BNIP3 contributes to the ability of KLF5 overexpression to restrain mtDNA‐sensitive inflammatory signaling.

Because the preceding experiments linked inflammatory activation to OM‐induced VIC mineralization, we next examined whether BNIP3 contributed to the mineralizing response modulated by KLF5. In KLF5‐overexpressing VICs, BNIP3 knockdown increased ALP and BMP2 expression (Figure 6Q) and partially reversed the reduction in calcium deposition, as shown by Alizarin Red staining (Figure 6R). These data indicate that BNIP3 knockdown markedly attenuated the reduction in mineralization associated with KLF5 overexpression under OM conditions.

To determine whether BNIP3 restoration could mitigate the consequences of KLF5 deficiency, BNIP3 was reintroduced into KLF5‐deficient VICs. BNIP3 restoration reduced ISG induction in KLF5‐deficient VICs under OM treatment (Figure 6S). Western blot analysis confirmed BNIP3 restoration and showed reduced STING and NLRP3 expression under KLF5‐deficient conditions (Figure 6T). Consistently, BNIP3 restoration decreased IL‐1β and TNF‐α secretion (Figure 6U), reduced ALP and BMP2 protein expression (Figure 6V), and attenuated matrix mineralization (Figure 6W). These reciprocal loss‐ and gain‐of‐function experiments identify BNIP3 as an important mediator of KLF5‐regulated mitochondrial quality control, mtDNA‐sensitive inflammatory signaling, and OM‐induced mineralization in VICs.

Finally, BNIP3 expression was also reduced in calcified human aortic valve tissues (Figure S10A,B), indicating that reduced BNIP3 expression is present in human CAVD. Together, these data support a model in which KLF5‐regulated BNIP3 expression contributes to mitochondrial quality control through mitophagy, thereby limiting cytosolic mtDNA accumulation, STING/NLRP3‐associated inflammatory signaling, and OM‐induced mineralization in VICs.

### 3.7 Spatial Mapping Links KLF5‐Low Disease‐Associated VIC Program to Inflammatory–Osteogenic Valve Niches

**FIGURE 7:**
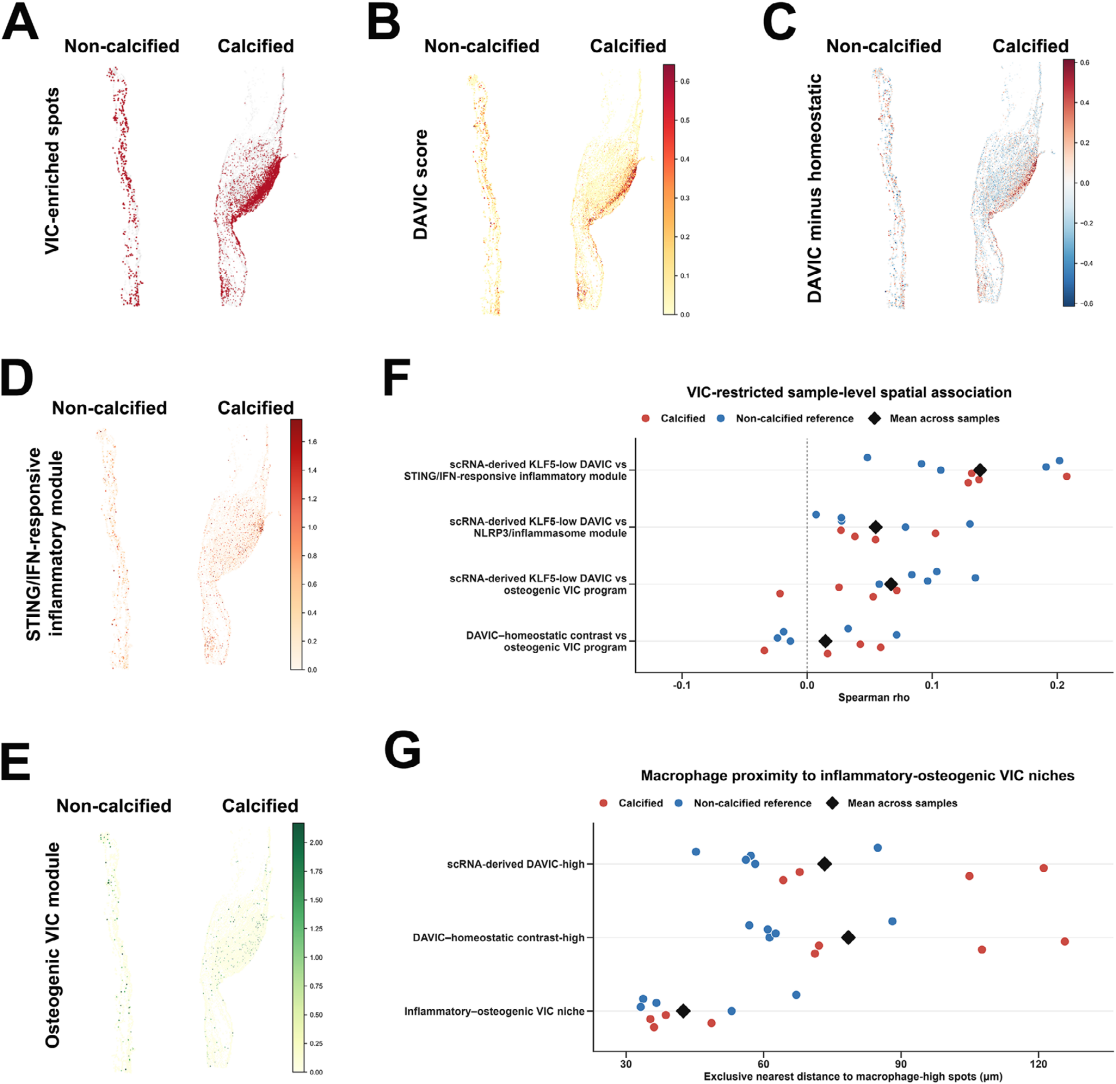
Spatial mapping links KLF5‐low disease‐associated VIC program to inflammatory–osteogenic valve niches. (A) Spatial mapping of VIC‐enriched spots in representative non‐calcified reference and calcified human aortic valve sections. (B) Spatial projection of the human scRNA‐derived KLF5‐low disease‐associated VIC (DAVIC) score onto post‐QC Stereo‐seq spots. (C–E) Spatial distributions of the DAVIC–homeostatic contrast score (C), STING/IFN‐responsive inflammatory module score (D), and osteogenic VIC module score (E). (F) VIC‐restricted sample‐level spatial association between the KLF5‐low DAVIC score or DAVIC–homeostatic contrast and inflammatory or osteogenic VIC‐related programs. Spearman's ρ is shown; each dot represents one spatial sample, and black diamonds indicate the mean across samples. (G) Macrophage proximity analysis showing the spatial‐library‐level summarized nearest distance from the indicated VIC niches to macrophage‐high regions. Each point represents one spatial library; shorter distances indicate greater proximity. Spatial transcriptomic analysis included four calcified/CAVD and five non‐calcific comparator libraries, retaining 66,960 spatial units after quality‐control filtering.

The spatial transcriptomic analysis included nine Stereo‐seq cell‐bin libraries, comprising four calcified/CAVD libraries and five non‐calcific reference libraries. After quality‐control filtering, 66,960 spatial units were retained, including 57,284 units from calcified/CAVD libraries and 9,676 units from comparator libraries. Representative spatial maps are shown for selected valve sections, whereas all nine libraries were included in spatial‐library‐level summary analyses. To determine whether the KLF5‐low VIC state defined at single‐cell resolution was spatially represented within human valve lesions, we analyzed human aortic valve Stereo‐seq data and first mapped VIC‐enriched spatial spots as the interstitial‐cell backbone for subsequent niche‐level analysis (Figure 7A). The KLF5‐low disease‐associated VIC (DAVIC) score was calculated by projecting the human scRNA‐derived KLF5‐low DAVIC signature onto post‐QC spatial spots, rather than by using raw KLF5 expression.

Projection of the human scRNA‐derived KLF5‐low DAVIC signature revealed higher DAVIC scores in calcified valve samples than in non‐calcified reference samples, with signal localization within VIC‐enriched regions of diseased tissue (Figure 7B). To distinguish disease‐associated VIC‐state activity from the homeostatic VIC background, we also calculated a DAVIC–homeostatic contrast score. This contrast highlighted lesion‐associated regions in the calcified valve section, whereas the non‐calcified reference section showed comparatively limited positive contrast (Figure 7C).

STING/IFN‐responsive inflammatory and osteogenic VIC module signals were detected within VIC‐enriched regions of calcified valve sections (Figure 7D,E). At the spatial‐library level, VIC‐restricted analyses showed positive associations between the KLF5‐low DAVIC score and STING/IFN‐responsive inflammatory, NLRP3/inflammasome, and osteogenic VIC programs (Figure 7F). The DAVIC–homeostatic contrast was included as an additional sensitivity readout for association with the osteogenic VIC program.

To further characterize the inflammatory microenvironment surrounding these VIC niches, we performed an exploratory macrophage proximity analysis. Nearest‐distance analysis showed that inflammatory–osteogenic VIC niches were closer to macrophage‐high regions than DAVIC‐high or DAVIC–homeostatic contrast‐high regions alone (Figure 7G). Although this analysis does not establish recruitment causality, the observed spatial proximity is consistent with an inflammatory microenvironment surrounding disease‐enriched VIC niches.

In summary, these spatial transcriptomic data localize the human scRNA‐derived KLF5‐low DAVIC signature to VIC‐rich inflammatory–osteogenic regions in calcified valves.

## 4 Discussion

In this study, we identify KLF5 as a regulator of stress‐responsive VIC remodeling in CAVD. Human tissue and scRNA‐seq analyses showed reduced KLF5 expression in VIC‐rich regions and selected remodeling/stress‐associated VIC states. Functional and rescue experiments in primary human VICs identified BNIP3 as an important mediator through which KLF5 maintains mitochondrial quality control and limits cytosolic mtDNA accumulation and downstream mtDNA‐sensitive inflammatory signaling. Spatial transcriptomics localized the scRNA‐derived KLF5‐low DAVIC program to VIC‐rich regions with inflammatory and osteogenic signatures in calcified valves.

CAVD is an active fibro‐inflammatory and osteogenic disease rather than a passive degenerative process [41]. VICs contribute to this progression by acquiring activated, inflammatory, matrix‐remodeling, and osteogenic states under pathological stress [7, 42]. KLF5 reduction was concentrated in remodeling/stress‐associated VIC states rather than uniformly distributed across the VIC compartment. Consistent with this interpretation, KLF5 manipulation altered the magnitude of OM‐induced osteogenic responses but did not induce appreciable mineralization under basal conditions. This stress‐dependent behavior differs from the reported pro‐calcific activity of KLF5 in phosphate‐treated vascular smooth muscle cells [31] and is consistent with the context‐dependent functions of KLF5 in cardiovascular cells [29, 43, 44]. Cell identity, chromatin state, extracellular matrix, biomechanical exposure, and the nature of the inducing stimulus may all influence KLF5 transcriptional output. The present data therefore indicate that KLF5 acts in a stress‐dependent manner in VICs rather than exerting a universal pro‐ or anti‐calcific effect.

KLF5 loss increased cytosolic mtDNA accumulation and enhanced TBK1, IRF3, and p65 activation in VICs. siRNA‐mediated cGAS depletion reduced these responses and attenuated ISG induction, while mtDNA depletion produced concordant effects. These findings place cytosolic mtDNA sensing through cGAS upstream of the amplified inflammatory response. This interpretation is consistent with evidence linking mitochondrial injury, mtDNA release, and STING activation to cardiovascular inflammation and valvular calcification [18]. KLF5‐deficient VICs also showed increased NLRP3, cleaved caspase‐1, IL‐1β, and TNF‐α under OM conditions. p65 knockdown reduced these responses, and p65 ChIP showed increased occupancy at the NLRP3 promoter after KLF5 depletion, consistent with NF‐κB‐dependent transcriptional priming of NLRP3 [45]. NLRP3 inflammasome activation has been implicated in calcific valve pathology [12]. Pharmacological STING inhibition with H‐151 attenuated p65 phosphorylation, NLRP3‐associated signaling, cytokine release, and OM‐induced mineralization, implicating STING in this response without establishing a genetic requirement for STING.

BNIP3‐mediated mitophagy is one mechanism through which KLF5 maintains mitochondrial quality control in VICs. Impaired autophagy and mitophagy have been reported in calcific aortic valve stenosis and associated with disease severity [25], while BNIP3 has established roles in cardiovascular stress adaptation [46]. Mitophagy limits inflammatory DNA sensing by removing dysfunctional mitochondria before mtDNA accumulates in the cytosol [21, 47]. Promoter‐reporter assays, motif mutagenesis, and KLF5 ChIP indicated direct transcriptional activation of BNIP3 by KLF5. Histone‐mark and 3C‐qPCR analyses provided complementary evidence of promoter‐associated chromatin changes after KLF5 depletion. Functionally, BNIP3 knockdown substantially attenuated the ability of KLF5 overexpression to preserve mitochondrial function, limit cytosolic mtDNA accumulation, suppress STING/NLRP3‐associated inflammatory signaling, and reduce OM‐induced mineralization. Conversely, BNIP3 re‐expression mitigated the inflammatory and mineralizing phenotypes associated with KLF5 deficiency. These findings identify BNIP3 as an important mediator of KLF5‐regulated mitochondrial quality control in VICs, while indicating that additional KLF5‐regulated stress‐response programs may also contribute to the observed phenotypes.

Systemic AAV2‐mediated KLF5 overexpression was associated with improved valvular hemodynamic indices and reduced leaflet thickening, mineral deposition, and BMP2 expression in ApoE^−/−^ mice. These observations provide supportive in vivo evidence that increased KLF5 expression modifies hyperlipidemia‐associated valve remodeling, but systemic delivery does not identify the cellular compartment responsible for the phenotype. Spatial transcriptomic analysis provided complementary localization in human tissue. Because KLF5 is a low‐abundance transcription factor and can be sparsely detected in single‐cell and spatial data, we projected a DAVIC state signature rather than relying on raw KLF5 expression alone. Spatial mapping localized this signature to VIC‐rich regions with inflammatory and osteogenic features and showed modest positive associations with STING/IFN‐responsive, NLRP3/inflammasome, and osteogenic VIC‐related modules. Macrophage proximity analysis was consistent with an inflammatory microenvironment surrounding these VIC niches. These findings provide tissue‐level localization but do not establish macrophage recruitment, direct cell–cell signaling, or causal interactions.

Current management of severe calcific aortic stenosis relies on surgical or transcatheter valve replacement [2], and no approved pharmacological therapy has been shown to halt disease progression [3, 48]. Because mineral deposition is a late, convergent feature of several pathological processes, interventions directed solely at calcification may not address upstream disease mechanisms. By identifying BNIP3‐mediated mitophagy as one mechanism linking KLF5 loss to cytosolic mtDNA accumulation and inflammatory signaling, our findings support further investigation of mitochondrial quality control as a potential upstream therapeutic target in CAVD. Whether this pathway can be targeted safely and selectively to modify disease progression remains to be determined.

Several limitations should be acknowledged. First, the human comparator tissues were non‐calcified reference valves obtained from Bentall procedures or heart transplantation cases, rather than fully healthy individuals. Second, bulk valve assays contain mixed cellular compartments. Serial histology, Vimentin/KLF5 co‐immunofluorescence, public human scRNA‐seq reanalysis, primary human VIC experiments, and spatial transcriptomics improved cell‐type attribution but did not eliminate this limitation. Third, the public bulk‐transcriptomic screen was designed as a focused cross‐cohort discovery analysis rather than an exhaustive meta‐analysis of all available CAVD datasets. Differences in platform, disease stage, valve morphology, tissue sampling, comparator definition, and sample size limit direct pooling and formal meta‐analysis across public CAVD cohorts. The public datasets were therefore used for discovery, followed by validation in an independent human valve cohort. In addition, AR samples in the public scRNA‐seq dataset served as non‐calcific disease comparators rather than healthy controls, and the spatial analyses established lesion‐associated localization and statistical associations rather than causal cell–cell interactions.

The in vivo findings also require cautious interpretation. AAV2 was administered systemically, and transgene expression was not restricted to VICs; therefore, contributions from endothelial cells, immune cells, liver, or other extra‐valvular tissues cannot be excluded. Although no significant differences were detected in the measured lipid, cardiac, hepatic, or renal parameters between HFD+AAV‐scr and HFD+AAV‐KLF5 mice, unmeasured systemic or non‐cell‐autonomous effects remain possible. The ApoE^−/−^ HFD model reproduces selected features of lipid‐associated valve remodeling but not the full chronic course or mechanical complexity of human CAVD. The animal experiments therefore complement the primary human VIC perturbation and rescue experiments, which provide the principal evidence for a VIC‐intrinsic mechanism. KLF5 is also a pleiotropic transcription factor. The present study identifies BNIP3 as an important mediator of the mitochondrial and inflammatory phenotypes examined here; additional KLF5‐regulated pathways remain to be defined in broader perturbation studies and cell‐type‐specific models.

In summary, KLF5 loss in VICs weakens BNIP3‐mediated mitophagy, increases cytosolic mtDNA accumulation, and amplifies STING/NF‐κB–NLRP3 inflammatory signaling under osteogenic stress. Findings from human tissues, primary VIC, animal, and spatial transcriptomic analyses link impaired KLF5‐regulated mitochondrial quality control to inflammatory and osteogenic valve remodeling. Therapeutic feasibility, cell‐selective targeting, and efficacy remain to be established, but these findings provide an upstream mechanistic rationale for future therapeutic investigation in CAVD.

## Author Contributions

JHB and YFS conceived and designed the experiments; JHB, JXG, CZY, XFX, and YQH performed the experiments; XC, ZY, and CZY analyzed the data and provided bioinformatics analysis; AL, HBJ, HSC, and YQH visualized the data; MLC, YFJ, YZ, and HBJ collected the clinical data; JHB and YFS wrote the manuscript; YFS provided technical and financial support. All authors read and approved the final manuscript.

## Funding

This work was supported by National Natural Science Foundation of China (82370376, 82570435), National Key Specialty Construction Project 2024 (CZ1320240102), Jiangsu Province Capability Improvement Project through Science, Technology and Education (ZDXK202230), Jiangsu Joint Project of Ili and Jiangsu Joint Institute of Health (yl2023ms11), Noncommunicable Chronic Diseases‐National Science and Technology Major Project (2024ZD0527200), and Young Scholars Fostering Fund of the First Affiliated Hospital of Nanjing Medical University (PY2025001).

## Ethics Statement

All experiments involving animals were conducted according to the ethical policies and procedures approved by the Institutional Animal Care and Use Committee of Nanjing Medical University (ethical approval number: IACUC‐2210022). Human tissue collection and all human‐related procedures were approved by the Ethics Committee of the First Affiliated Hospital of Nanjing Medical University (2019‐SRFA‐280), and written informed consent was obtained from all participants.

## Conflicts of Interest

The authors declare no conflicts of interest.

## Supporting information




**Supporting File**: advs77368‐sup‐0001‐SuppMat.docx.

## Data Availability

Public datasets analyzed in this study are available from GEO under accession numbers GSE235995, GSE153555, GSE83453, GSE291890, and GSE154513. Additional data generated in this study are available from the corresponding author upon reasonable request.
