# Supplementary material for: KLF5 Downregulation Links Impaired BNIP3‐Mediated Mitophagy to Inflammatory Valve Remodeling in Calcific Aortic Valve Disease
Source: Adv Sci (Weinh). 2026 Aug 27;13(63):e77368. doi: 10.1002/advs.77368 (PMC13521178; doi:10.1002/advs.77368)
Supplement: Supplementary file 1 [file ADVS-13-e77368-s001.docx]

**Supplemental Material**

**Supplemental Methods**

**Public GEO datasets and KLF-focused differential expression**

Public transcriptomic datasets of human aortic valve tissues were obtained from GEO. GSE235995, GSE153555, and GSE83453 were used as the primary KLF-family discovery cohorts because they provided donor-level phenotype annotations permitting defined disease–reference comparisons, included independent biological replication, and provided gene-level expression data covering KLF-family genes. Each cohort was analyzed separately to avoid direct cross-platform pooling and associated batch effects. Samples were assigned to CAVD or non-CAVD reference/comparator groups according to the original GEO phenotype annotations. For RNA-seq count datasets (GSE235995 and GSE153555), raw counts were normalized using the trimmed mean of M-values (TMM) method and log-transformed prior to differential testing. For GSE83453, the primary comparison was restricted to stenotic and non-stenotic tricuspid aortic valves; bicuspid aortic valve samples were excluded from this comparison. The GEO-provided processed expression matrix was analyzed without additional transformation.

Differential expression analysis was restricted to KLF1–KLF17/18. *P* values were adjusted by the Benjamini–Hochberg procedure. KLF genes meeting nominal *p* < 0.05 and within-family FDR < 0.10 in an individual cohort were considered differentially expressed. Significant KLF-family members were intersected across the three discovery cohorts to identify shared dysregulated candidates.

**Histological processing and quantitative analysis of aortic valves**

Mouse hearts were harvested and perfused with 4% paraformaldehyde. The basal portion of the heart containing the aortic root was embedded in paraffin with a standardized orientation using anatomical landmarks to ensure that serial cross-sections were cut perpendicular to the longitudinal axis of the aortic root, allowing for the simultaneous analysis of all three aortic valve cusps. Serial sectioning (5 μm thickness) was initiated at the level where the valve commissures first appeared. To avoid sampling bias and recounting the same anatomical area, every fifth section was collected and stained, resulting in the analysis of a minimum of five non-adjacent, evenly spaced sections per mouse covering the majority of the valve cusp length. Valve thickness was quantified at the nodule of Arantius in the middle of each leaflet. The overall thickness for each valve was defined as the average of the three largest leaflet thicknesses measured across the five sections.

**Extra-mitochondrial DNA^+^ foci quantification**

Images were background-subtracted (rolling ball 25 pixels) and lightly smoothed. Single-cell ROIs were drawn manually. Nuclei were segmented from the PicoGreen channel and excluded to define the cytoplasm. A mitochondrial mask was generated from the MitoTracker channel. Extra-mitochondrial DNA was defined as PicoGreen signal within the cytoplasm that did not overlap with the MitoTracker mask. DNA^+^ foci were quantified per cell in ImageJ, with uniform thresholds applied to all images.

**Enzyme-linked immunosorbent assay (ELISA)**

Culture supernatants were collected at the indicated time points, centrifuged (1,000 g, 20 min, 4 °C) to remove debris, aliquoted, and stored at −80 °C (single freeze–thaw). IL-1β and TNF-α were quantified using commercial ELISA kits (Abclonal) following the manufacturer’s protocol. Samples and standards were run in duplicate; absorbance was read at 450 nm with 570 nm reference subtraction.

**Hematoxylin and Eosin (H&E) Staining**

Aortic valve leaflets were fixed in 4% paraformaldehyde, embedded in paraffin, and cut into 5 μm-thick serial sections. Following deparaffinization in xylene and rehydration through a descending ethanol gradient, the sections were stained with hematoxylin, differentiated, and counterstained with eosin. After rapid dehydration and clearing in xylene, the slides were mounted with neutral resin. Representative histological images were then captured using an optical microscope.

**Calcium content determination**

Calcium content was determined colorimetrically using the o-cresolphthalein complexone method according to the manufacturer’s instructions (Beyotime Biotech, Shanghai, China). Total cellular protein was quantified using a BCA Protein Assay Kit (Vazyme Biotech, Nanjing, China) to normalize the calcium levels. The results are expressed as micrograms of calcium per milligram of total protein (μg/mg protein).

**Detection of mitochondrial membrane potential**

Mitochondrial membrane potential was assessed using the JC-1 Assay Kit (Beyotime Biotechnology, C2003S) as per the manufacturer's instructions. VICs were incubated with JC-1 working solution at 37°C for 20 min. Fluorescence imaging was performed using a fluorescence microscope. JC-1 aggregates emit red fluorescence at high membrane potentials, while JC-1 monomers emit green fluorescence at lower membrane potentials. The red-to-green fluorescence ratio was used as an index of mitochondrial membrane potential; a lower ratio indicates mitochondrial depolarization.

**Public VIC epigenomic analysis and BNIP3 regulatory-region nomination**

Processed public human VIC epigenomic data from GSE154513 were used to contextualize the experimentally validated KLF5–BNIP3 promoter interaction and to nominate candidate BNIP3 promoter-associated contacts. The analyzed tracks included ATAC-seq, H3K27ac ChIP-seq, H3K4me3 ChIP-seq, H3K4me1 ChIP-seq, and H3K27ac HiChIP. All analyses were performed on the hg19 genome build using GENCODE v19 annotation. Processed bigWig tracks were used for signal visualization and target-region selection only, not for peak calling or differential analysis. The P1 interval was selected as a motif-proximal promoter region overlapping or closely flanking the validated KLF5-responsive motif and was used for H3K27ac ChIP-qPCR. The P2 interval was selected as a TSS-proximal promoter region and was used for H3K27ac and H3K4me3 ChIP-qPCR. In addition, candidate BNIP3 promoter-associated contacts were nominated from public H3K27ac HiChIP data. Candidate distal regions C1–C3 were prioritized based on HiChIP contact support to the BNIP3 promoter anchor, H3K27ac/H3K4me1-marked chromatin context, genomic distance, and 3C-qPCR assay feasibility.

**Public single-cell RNA-seq analysis**

Public human aortic valve single-cell RNA-seq data were obtained from GEO (GSE291890). Seven non-sorted scRNA-seq Flex libraries from six patients were analyzed, including two AR patients represented by one library each and four AS patients represented by five libraries in total; one AS patient was represented by two libraries. AR samples were analyzed as non-calcific aortic regurgitation disease comparators rather than healthy controls. After quality-control filtering, 18,565 cells were retained, including 16,877 AR cells and 1,688 AS cells. Count matrices and available metadata were processed using Seurat. Cells passing quality-control filtering were normalized, subjected to principal component analysis, clustered, and visualized by UMAP. Post-QC distributions of nFeature_RNA, nCount_RNA, and mitochondrial transcript percentage across libraries are shown in Figure S1A. Major valvular cell compartments, including VICs, VECs, macrophage/monocyte cells, lymphoid cells, dendritic cells, and other minor populations, were annotated using canonical marker genes and available dataset annotations. Rare or low-confidence immune populations were conservatively grouped as Other. For major-cell comparisons, KLF5 detection fraction was calculated within each library as the proportion of cells with non-zero KLF5 RNA counts. Only library–cell-type strata containing at least 20 cells were included in the AS–AR comparison. Differences between eligible library-level estimates were summarized with 95% confidence intervals obtained from 5,000 bootstrap resamples. KLF5 detection and mean normalized expression across major cell populations were additionally displayed separately for AR and AS as a descriptive cell-type distribution analysis. VICs were then subsetted and reclustered to resolve VIC-state heterogeneity. VIC states were annotated according to state-enriched marker genes and included homeostatic, quiescent, activated-remodeling, inflammatory-stress, stromal-complement, osteogenic, and interferon-response VIC states. KLF5 detection fraction was calculated as the proportion of cells with non-zero KLF5 RNA counts at the patient/library and VIC-state levels. KLF5-low disease-associated VIC states were defined after comparison with homeostatic VICs and were used to generate the DAVIC signature for downstream spatial projection.

**Spatial transcriptomic analysis**

Human aortic valve spatial transcriptomic data were analyzed to determine whether the scRNA-derived KLF5-low DAVIC program was spatially represented within calcified valve lesions. The analysis included nine Stereo-seq cell-bin libraries, comprising four calcified/CAVD libraries and five non-calcified reference libraries. After quality-control filtering, 66,960 cell-bin spatial units were retained, including 57,284 units from calcified/CAVD libraries and 9,676 units from non-calcified reference libraries. For consistency, these retained cell-bin units are referred to as spatial spots throughout the manuscript. Spatial spots passing quality-control filtering were normalized and scored for major cell-type and functional gene modules. VIC-enriched spots were identified using scRNA-derived VIC marker signatures and were used as the interstitial-cell backbone for VIC-restricted spatial analyses. The KLF5-low DAVIC score was calculated by projecting the scRNA-derived KLF5-low DAVIC signature onto post-QC spatial spots using a module-score approach. A homeostatic VIC score was computed in parallel, and the DAVIC–homeostatic contrast score was defined as the difference between the DAVIC score and the homeostatic VIC score. STING/IFN-responsive inflammatory, NLRP3/inflammasome, and osteogenic VIC module scores were calculated using predefined gene sets consistent with the cell-based mechanisms examined in this study. VIC-restricted spatial association analyses were performed using spatial-library-level summaries and Spearman correlation. For macrophage proximity analysis, macrophage-high regions were defined using macrophage marker/module scores, and nearest-distance measurements from inflammatory–osteogenic VIC niches to macrophage-high spots were calculated using spatial coordinates.

**Von Kossa staining**

Aortic valve tissue mineralization was assessed using Von Kossa staining. Sections were incubated in 5% silver nitrate solution, exposed to strong light for 30 min, and treated with 5% sodium thiosulfate for 3 min to develop the calcium deposits. The stained sections were examined and imaged using an optical microscope. ImageJ software was used for quantitative analysis of the mineralization.

**Supplementary Figures S1-10**

**Figure S1. Quality control, cell annotation, and KLF5 distribution in public human aortic valve scRNA-seq data.**

(**A**) Post-QC distributions of detected genes, UMI counts, and mitochondrial transcript percentage across the seven analyzed scRNA-seq Flex libraries.

(**B**) Canonical marker-gene expression supporting the annotation of VICs, VECs, macrophage/monocyte cells, lymphoid cells, dendritic cells, and other minor populations. Dot size indicates the proportion of cells with detectable expression, and color indicates gene-wise scaled average expression. PTPRC is shown as a pan-immune marker.

(**C**) KLF5 detection and mean normalized expression across major valve cell populations in AR and AS. Dot size indicates the proportion of cells with detectable KLF5 RNA counts, and color indicates mean normalized KLF5 expression. AR samples represent non-calcific aortic regurgitation disease comparators rather than healthy controls.

**Figure S2. Definition and transcriptomic annotation of the KLF5-low DAVIC signature.**

(**A**) Dot plot showing detection fraction and average expression of representative KLF5-low DAVIC signature genes across VIC states.

(**B**) Exploratory gene-set enrichment analysis comparing strict KLF5-low DAVIC states with homeostatic VICs. Positive normalized enrichment scores indicate gene sets directionally enriched in strict KLF5-low DAVIC states, whereas negative scores indicate gene sets directionally enriched in homeostatic VICs.

**Figure S3. AAV2-mediated KLF5 expression in mouse aortic valve leaflets.**

(**A**) Immunofluorescence staining showing co-localization of KLF5 and the VIC marker vimentin (n = 6 individual mice per group; scale bar, 50 μm in overview images and 10 μm in enlarged views of the boxed regions).

(**B**) Quantification of KLF5 fluorescence intensity in aortic valve leaflets from the indicated groups. Statistical significance is indicated as ****p* < 0.001.

**Figure S4. KLF5 expression in the liver and kidney after systemic AAV2 delivery.**

(**A and B**) qRT-PCR analysis of KLF5 mRNA levels in liver and kidney tissues of mice (n = 6 individual mice per group).

(**C**) IHC staining of KLF5 expression levels in liver tissues from different groups (n = 6 individual mice per group; scale bar, 100 μm in overview images and 50 μm in enlarged views of the boxed regions).

Statistical significance is indicated as ****p* < 0.001, ns indicates no statistical significance.

**Figure S5. cGAS knockdown attenuates KLF5-deficiency-enhanced DNA-sensing inflammatory signaling in VICs.**

**(A)** Western blot analysis of cGAS, STING, p-TBK1, p-IRF3/IRF3, and p-p65/p65 protein levels in OM-treated control and KLF5-deficient VICs transfected with si-NC or si-cGAS (n = 5 independent biological replicates per group).

**(B)** qRT-PCR measurement of IFIT1, IFI44, MX2, ISG15, and CXCL10 transcripts in the indicated groups (n = 5 independent biological replicates per group).

Statistical significance is indicated as ****p* < 0.001, ns indicates no statistical significance.

**Figure S6.** **Activation of the cGAS–STING pathway in human calcified aortic valve tissues.**

(**A**) GSEA enrichment analysis showing significant enrichment in interferon signaling, TNF-α signaling, and inflammatory response pathways in the GSE153555 dataset.

(**B**) Cytosolic mtDNA targets, nuclear LINE1 elements or 18S rRNA were measured by qPCR (n = 10 independent human valve specimens per group).

(**C**) Western blot analysis of cGAS, STING, p-TBK1, IRF3, p-IRF3, p65, and p-p65 protein levels (n = 10 independent human valve specimens per group).

(**D**) ISGs (IFIT1, IFI44, MX2, ISG15, and CXCL10) mRNA levels were measured by qRT-PCR (n = 10 independent human valve specimens per group).

Statistical significance is indicated as ****p* < 0.001, ***p* < 0.01, ns indicates no statistical significance.

**Figure S7. NLRP3 inflammasome activation in OM-treated VICs.**

(**A**) Western blot analysis of NLRP3, cleaved caspase-1, and IL-1β protein levels (n = 5 independent biological replicates per group).

(**B**) IL-1β and TNF-α mRNA levels were measured by qRT-PCR (n = 5 independent biological replicates per group).

(**C**) ELISA measurements of IL-1β and TNF-α concentrations in cell supernatants (n = 5 independent biological replicates per group).

Statistical significance is indicated as ****p* < 0.001, ***p* < 0.01.

**Figure S8. KLF5 upregulation promotes mitophagy and reduces ROS levels.**

(**A**) Mitochondrial membrane potential was evaluated by JC-1 staining (n = 6 independent biological replicates per group; scale bar = 50 μm).

(**B**) DHE staining was used to evaluate ROS levels in VICs (n = 6 independent biological replicates per group; scale bar = 50 μm).

(**C**) Representative TEM images showed mitochondrial ultrastructure and autophagic structures containing mitochondrial profiles (n = 6 independent biological replicates per group; scale bar = 500 nm).

(**D**) Confocal microscopy showed the colocalization of GFP-LC3 and mitochondria (n = 6 independent biological replicates per group; scale bar = 10 μm).

(**E**) Cytosolic mtDNA was measured by qPCR (n = 6 independent biological replicates per group).

Statistical significance is indicated as ****p* < 0.001, ***p* < 0.01, ns indicates no statistical significance.

**Figure S9. Effects of BNIP3 knockdown on ROS accumulation and p65 localization in KLF5-overexpressing VICs.**

(**A**) Representative DHE fluorescence images and quantification of ROS levels (n = 6 independent biological replicates per group; scale bar = 50 μm).

(**B**) Immunofluorescence staining of p65 in the indicated groups (n = 5 independent biological replicates per group; scale bar = 20 μm).

Statistical significance is indicated as ****p* < 0.001.

**Figure S10. Reduced BNIP3 expression in calcified human aortic valves.**

(**A**) Western blot analysis of BNIP3 expression levels in human aortic valves (n = 10 independent human valve specimens per group).

(**B**) Representative BNIP3 immunofluorescence staining and quantification in human aortic valve tissues (n = 10 independent human valve specimens per group; scale bar, 100 μm in overview images and 20 μm in enlarged views of the boxed regions).

Statistical significance is indicated as ***p* < 0.01.

**Tables S1-S3**

**Table S1.** **Clinical characteristics of patients providing non-calcified reference valves and patients with CAVD**

| **Parameters** | **Control (n=15)** | **CAVD (n=15)** | ***P* value** |
| --- | --- | --- | --- |
| Mean age (years) | 57.87 ± 5.10 | 60.27 ± 6.67 | 0.279 |
| Male | 11 | 12 | 1.000 |
| Hypertension | 6 | 8 | 0.715 |
| Hypercholesterolemia | 7 | 5 | 0.710 |
| Diabetes mellitus | / | / | / |
| Heart failure | / | / | / |
| Chronic kidney disease | / | / | / |
| ACEI/ARB | 6 | 7 | 1.000 |
| β-blockers | 7 | 8 | 1.000 |
| Statins | 7 | 5 | 0.710 |
| Smoking | 4 | 6 | 0.700 |
| LVEF (%) | 57 ± 7.7 | 56 ± 8.1 | 0.732 |
| Transvalvular pressure  gradient (mmHg) | 13.87 ± 3.78 | 38.93 ± 11.95 | < 0.001 |
| AVA (cm^2^) | / | 0.90 ± 0.29 | / |
| ALT (U/L) | 28.2 ± 9.8 | 30.7 ± 10.9 | 0.514 |
| AST (U/L) | 20.4 ± 5.7 | 24.1 ± 6.2 | 0.100 |
| TC (mmol/L) | 3.45 ± 1.31 | 3.52 ± 1.24 | 0.882 |
| TG (mmol/L) | 1.84 ± 0.85 | 2.01 ± 1.14 | 0.647 |
| LDL (mmol/L) | 2.93 ± 1.54 | 3.26 ± 1.79 | 0.593 |
| Cr (μmol/L) | 65.5 ± 14.9 | 70.1 ± 16.8 | 0.434 |
| BUN (mmol/L) | 5.47 ± 0.49 | 5.68 ± 0.62 | 0.312 |

Control denotes patients providing non-calcified reference valves; CAVD, calcific aortic valve disease; ACEI, angiotensin-converting enzyme inhibitor; ARB, angiotensin receptor blocker; LVEF, left ventricular ejection fraction; AVA, aortic valve area; ALT, alanine aminotransferase; AST, aspartate aminotransferase; TC, total cholesterol; TG, triglyceride; LDL, low-density lipoprotein cholesterol; Cr, serum creatinine; BUN, blood urea nitrogen. Values are mean ± standard deviation (SD). Statistical comparisons for continuous variables were performed using a two-tailed unpaired t-test. Categorical variables were compared using Fisher’s exact test.

**Table S2. List of primers applied for this research**

| **Primers** | **Primer sequence (5′ to 3′)** |
| --- | --- |
| KLF5 forward | TCAGTCGTAGACCAGTTCTTCA |
| KLF5 reverse | CTGGGATTTGTAGAGGCCAGT |
| BMP2 forward | ACCCGCTGTCTTCTAGCGT |
| BMP2 reverse | TTTCAGGCCGAACATGCTGAG |
| ALP forward | AACATCAGGGACATTGACGTG |
| ALP reverse | GTATCTCGGTTTGAAGCTCTTCC |
| β-actin forward | CTACCTCATGAAGATCCTCACCGA |
| β-actin reverse | TTCTCCTTAATGTCACGCACGATT |
| MT-ND1 forward | CTCTTCGTCTGATCCGTCCT |
| MT-ND1 reverse | TGAGGTTGCGGTCTGTTAGT |
| MT-ND2 forward | GTAGACAGTCCCACCCTCAC |
| MT-ND2 reverse | TTGATCCCGTTTCGTGCAAG |
| L1ORF1 forward | AGAACGCCACAAAGATACTCCTCG |
| L1ORF1 reverse | CTCTCTTCTGGCTTGTAGGGTTTCTG |
| L1ORF2 forward | AAACTGAACAACCTGCTCCTGAATG |
| L1ORF2 reverse | CTACACACTGCTTTGAATGCGTCC |
| RNA18S forward | GTAACCCGTTGAACCCCATT |
| RNA18S reverse | CCATCCAATCGGTAGTAGCG |
| BNIP3 forward | CAGGGCTCCTGGGTAGAACT |
| BNIP3 reverse | CTACTCCGTCCAGACTCATGC |
| ISG15 forward | CACCTGAAGCAGCAAGTGAG |
| ISG15 reverse | ATTTCCGGCCCTTGATCCTG |
| CXCL10 forward | GTGGCATTCAAGGAGTACCTC |
| CXCL10 reverse | TGATGGCCTTCGATTCTGGATT |
| MX2 forward | GGGGACAGGACCATCGGTAT |
| MX2 reverse | TTTCTTGGTTGCCTCTGCCA |
| IFIT1 forward | TTGATGACGATGAAATGCCTGA |
| **Primers** | **Primer sequence (5′ to 3′)** |
| IFIT1 reverse | CAGGTCACCAGACTCCTCAC |
| IFI44 forward | TTTTCGATGCGAAGATTCACTGG |
| IFI44 reverse | CCTGATGCGTTACATGCCCTT |
| IL-1β forward | TTGCCAGCCAGTGACACAAT |
| IL-1β reverse | GAGAAGGTGGTTGTCTGGGAAT |
| TNF forward | CCTCTCTCTAATCAGCCCTCTG |
| TNF reverse | GAGGACCTGGGAGTAGATGAG |

**Table S3.** **Echocardiographic and metabolic parameters in different groups of mice.**

| **Parameters** | **ND+AAV-scr**  **(n = 6)** | **ND+AAV-KLF5**  **(n = 6)** | **HFD+AAV-scr**  **(n = 6)** | **HFD+AAV-KLF5**  **(n = 6)** |
| --- | --- | --- | --- | --- |
| Body weight (g) | 29.5 ± 3.39 | 31.08 ± 3.37 | 38.68 ± 3.26 | 40.32 ± 3.41 |
| Heart rate (bpm) | 514.69 ± 27.26 | 510.77 ± 30.41 | 523.21 ± 29.87 | 533.64 ± 31.32 |
| LVPWd (mm) | 1.33 ± 0.19 | 1.42 ± 0.25 | 1.51 ± 0.24 | 1.44 ± 0.17 |
| LVEDd (mm) | 3.22 ± 0.39 | 3.15 ± 0.44 | 3.18 ± 0.54 | 3.14 ± 0.49 |
| IVSd (mm) | 0.75 ± 0.12 | 0.83 ± 0.14 | 0.95 ± 0.16 | 0.85 ± 0.13 |
| EF (%) | 75.05 ± 5.54 | 74.25 ± 4.72 | 72.34 ± 6.13 | 75.81 ± 6.52 |
| FS (%) | 51.5 ± 3.65 | 49.37 ± 3.42 | 52.03 ± 3.18 | 50.52 ± 3.87 |
| Glucose (mmol/L) | 10.05 ± 2.08 | 9.87 ± 1.93 | 10.27 ± 1.81 | 9.96 ± 1.77 |
| TC (mmol/L) | 10.15 ± 2.28 | 11.29 ± 2.75 | 22.15 ± 5.43 | 21.28 ± 4.56 |
| LDL (mmol/L) | 8.34 ± 2.87 | 7.84 ± 2.69 | 19.53 ± 2.94 | 18.67 ± 3.16 |
| TG (mmol/L) | 1.78 ± 0.81 | 1.61 ± 0.75 | 3.53 ± 0.89 | 3.67 ± 0.68 |
| ALT (U/L) | 35.21 ± 6.27 | 38.54 ± 5.32 | 155.62 ± 19.54 | 162.37 ± 20.35 |
| AST (U/L) | 65.54 ± 8.92 | 68.32 ± 7.83 | 221.87 ± 25.42 | 230.49 ± 21.38 |
| BUN (mmol/L) | 8.72 ± 1.75 | 7.98 ± 1.89 | 9.09 ± 1.97 | 8.51 ± 1.76 |
| Cr (μmol/L) | 37.55 ± 4.87 | 35.04 ± 5.52 | 39.21 ± 4.73 | 37.54 ± 5.04 |

ND, normal diet; HFD, high-fat diet; LVPWd, left ventricular posterior wall diameter; LVEDd, left ventricular end diastolic diameter; IVSd, intraventricular septal diameter; EF, ejection fraction; FS, fractional shortening; TC, total cholesterol; LDL, low-density lipoprotein; TG, triglycerides; ALT, alanine aminotransferase; AST, aspartate aminotransferase; BUN, blood urea nitrogen; Cr, serum creatinine. Values are mean ± standard deviation (SD).
